# Hybrid HHO–WHO Optimized Transformer-GRU Model for Advanced Failure Prediction in Industrial Machinery and Engines

**DOI:** 10.3390/s26020534

**Published:** 2026-01-13

**Authors:** Amir R. Ali, Hossam Kamal

**Affiliations:** 1 Mechatronics Engineering Department, Faculty of Engineering and Materials Science (EMS), German University in Cairo (GUC), New Cairo 11835, Egypt; hosam.khalil@student.guc.edu.eg; 2 ARAtronics Laboratory, Mechatronics Engineering Department (MCTR), German University in Cairo (GUC), New Cairo 11835, Egypt

**Keywords:** failure time prediction, remaining useful life (RUL), automated hyperparameter tuning, hybrid HHO–WHO algorithm, transformer-GRU model, industrial machinery, engines, smart manufacturing

## Abstract

Accurate prediction of failure in industrial machinery and engines is critical for minimizing unexpected downtimes and enabling cost-effective maintenance. Existing predictive models often struggle to generalize across diverse datasets and require extensive hyperparameter tuning, while conventional optimization methods are prone to local optima, limiting predictive performance. To address these limitations, this study proposes a hybrid optimization framework combining Harris Hawks Optimization (HHO) and Wild Horse Optimization (WHO) to fine-tune the hyperparameters of ResNet, Bi-LSTM, Bi-GRU, CNN, DNN, VAE, and Transformer-GRU models. The framework leverages HHO’s global exploration and WHO’s local exploitation to overcome local optima and optimize predictive performance. Following hybrid optimization, the Transformer-GRU model consistently outperformed all other models across four benchmark datasets, including time-to-failure (TTF), intelligent maintenance system (IMS), C-MAPSS FD001, and FD003. On the TTF dataset, mean absolute error (MAE) decreased from 0.72 to 0.15, and root mean square error (RMSE) from 1.31 to 0.23. On the IMS dataset, MAE decreased from 0.04 to 0.01, and RMSE from 0.06 to 0.02. On C-MAPSS FD001, MAE decreased from 11.45 to 9.97, RMSE from 16.02 to 13.56, and score from 410.1 to 254.3. On C-MAPSS FD003, MAE decreased from 11.28 to 9.98, RMSE from 15.33 to 14.57, and score from 352.3 to 320.8. These results confirm that the hybrid HHO–WHO optimized Transformer-GRU framework significantly improves prediction performance, robustness, stability, and generalization, providing a reliable solution for predictive maintenance.

## 1. Introduction

In modern industrial systems, accurate prediction of failure time (FT) and remaining useful life (RUL) is crucial for minimizing unplanned downtime, reducing maintenance costs, and ensuring safe operations [1]. Machine and engine failures can lead to production losses, safety risks, and significant financial impacts. Consequently, predictive maintenance has become a critical research focus, aiming to forecast degradation patterns and anticipate failures before occurrence. In particular, machine learning and deep learning methods have shown strong potential in predictive maintenance by learning from sensor data to model machine and engine degradation and provide early failure warnings [2].

Despite these advances, existing predictive models continue to face significant limitations. Many approaches do not generalize well across datasets with different operating conditions [3]. Their performance also depends heavily on manual hyperparameter tuning, which requires expertise, increases computational cost, and reduces performance and scalability [4]. Furthermore, current models often fail to fully capture the nonlinear dependencies and long-term temporal dynamics in machine and engine signals, leading to reduced robustness and inconsistent performance. Conventional optimization methods are prone to local optima, which further limits predictive performance, especially across diverse industrial datasets.

To overcome these challenges, this research introduces a novel hybrid optimization framework combining Harris Hawks Optimization (HHO) and Wild Horse Optimization (WHO) for automated hyperparameter tuning. HHO is designed for global exploration, broadly searching the hyperparameter space to avoid local optima, while WHO is responsible for local exploitation, refining promising solutions to accelerate convergence toward optimal parameters. The integration of HHO and WHO balances exploration and exploitation, reducing dependence on manual parameter tuning and enhancing model performance stability, robustness, and generalization.

The proposed framework was applied to several state-of-the-art deep learning architectures, including Residual Network (ResNet), Bi-directional Long Short-Term Memory (Bi-LSTM), Bidirectional Gated Recurrent Unit (Bi-GRU), Convolutional Neural Network (CNN), Deep Neural Network (DNN), Variational Autoencoder (VAE), and Transformer Gated Recurrent Unit (Transformer-GRU). Among these, Transformer-GRU consistently outperformed other models due to the Transformer’s ability to capture long-range dependencies and the GRU’s effectiveness in modeling sequential patterns.

The models were evaluated on four widely used benchmark datasets: the time-to-failure (TTF) dataset, the intelligent maintenance system (IMS) dataset, and the C-MAPSS FD001 and FD003 datasets. Performance was assessed using mean absolute error (MAE) and root mean square error (RMSE). Following hybrid HHO–WHO optimization, Transformer-GRU achieved substantial improvements in predictive performance, robustness, and generalization. On the TTF dataset, MAE decreased from 0.72 to 0.15 and RMSE from 1.31 to 0.23. On the IMS dataset, MAE decreased from 0.04 to 0.01 and RMSE from 0.06 to 0.02. On the C-MAPSS FD001 dataset, MAE decreased from 11.45 to 9.97, RMSE from 16.02 to 13.56, and score from 410.1 to 254.3. On the C-MAPSS FD003 dataset, MAE decreased from 11.28 to 9.98, RMSE from 15.33 to 14.57, and score from 352.3 to 320.8.

These results confirm that the HHO–WHO hybrid framework effectively improves predictive performance. HHO ensures global exploration to avoid suboptimal solutions, while WHO accelerates local exploitation to fine-tune hyperparameters, collectively enhancing Transformer-GRU’s prediction capabilities across diverse datasets, including TTF, IMS, and C-MAPSS. This study bridges a critical gap in predictive failures by reducing reliance on manual tuning, improving model performance, and strengthening reliability. It establishes a robust, automated, and scalable predictive maintenance framework for industrial machinery and engines. The contributions offered by this research can be outlined as follows:

- Proposing a novel hybrid optimization framework by combining Harris Hawks Optimization (HHO) and Wild Horse Optimization (WHO) to establish a robust metaheuristic approach for automated hyperparameter tuning and enhanced predictive model performance.
- Leveraging HHO’s global exploration and WHO’s local exploitation within the framework to maintain a balanced exploration–exploitation trade-off, mitigate local optimum entrapment, and improve generalization, reducing reliance on manual configuration.
- Developing an optimized Transformer-GRU architecture that effectively captures temporal degradation patterns in industrial machinery and engines, improving failure prediction performance, robustness, stability, and generalization.
- Demonstrating and validating superior performance on the TTF, IMS, and C-MAPSS FD001 and FD003 datasets through comparison and ablation studies, confirming the effectiveness of the proposed hybrid framework and its consistent outperformance of baseline models.

The structure of this paper is arranged as follows: Section 2 reviews related work and identifies key research gaps. Section 3 details the proposed methodology, including model design and implementation. Section 4 presents the experimental setup and analyzes the results. Section 5 discusses encountered challenges and study limitations. Section 6 concludes the research and outlines potential future research directions.

## 2. Related Works

Accurate failure prediction and remaining useful life estimation have attracted considerable attention across industrial domains due to their potential in reducing unplanned downtimes, optimizing maintenance schedules, and improving overall operational efficiency. Various methodologies have evolved, ranging from traditional statistical methods and machine learning models to deep learning and optimization frameworks.

In modern industrial environments, particularly within smart manufacturing systems characterized by high levels of automation and integration, equipment reliability is essential for ensuring consistent and efficient operations. Rolling-element bearings (REBs) are critical mechanical components widely used in applications such as electric motors, turbines, and aircraft engines. Failures in these components often lead to secondary damage in adjacent parts, resulting in safety hazards, unexpected system downtime, and increased maintenance costs [5,6]. Consequently, predictive maintenance strategies have increasingly emphasized the importance of accurately estimating the remaining useful life of bearings to prevent catastrophic failures and avoid unnecessary interventions.

RUL refers to the predicted duration from the current point in time until a system or component reaches the end of its operational life [7]. In the context of bearings, this represents the time interval between the latest condition assessment and the expected onset of functional failure. Unlike mean time-to-failure (MTTF), which provides an average lifespan assuming non-repairable conditions, RUL offers a dynamic, condition-based estimate that depends on real-time health indicators and predefined end-of-life thresholds [8]. Bearing lifespans can vary significantly even under uniform operating conditions due to differences in material quality, installation performance, and load dynamics. This variability has been observed in several benchmark degradation datasets, including IMS, PROGNOSTIA, and XJTU-SY [9,10,11]. As a result, accurate RUL prediction is crucial for improving system reliability and supporting the implementation of data-driven maintenance strategies.

Rolling bearings play a vital role in numerous industrial and mechanical systems, including aerospace, engine systems, mining, and agriculture, where they are often referred to as “industrial joints” due to their essential function in enabling rotational motion. However, these components are susceptible to various failure mechanisms such as fatigue, wear, corrosion, electrical erosion, and structural damage, with material fatigue and inadequate lubrication identified as the most prevalent causes [12]. Preventive measures such as regular lubrication and operation within rated conditions have been shown to significantly reduce the risk of these failures [13]. Because bearing failures can compromise machinery reliability, leading to unplanned downtimes or total equipment breakdowns, they often result in both economic losses and safety risks [14,15]. Therefore, accurately predicting the remaining useful life of bearings is critical for enabling effective maintenance planning.

RUL is generally defined as the time from a bearing’s current health state until it reaches a defined failure threshold [16]. Prediction methods typically fall into two broad categories, including physics-based and data-driven approaches [17]. Physics-based models aim to replicate the physical degradation processes using mechanistic formulations, such as the Paris crack propagation model, the Forman fatigue crack growth model, and the Palmgren-Miner cumulative damage theory [18,19,20]. These models offer interpretability and require limited training data, but they often struggle with scalability and precision in complex, integrated systems. Consequently, recent advancements in sensor technology and artificial intelligence have steered focus toward data-driven methods [21].

Data-driven techniques are commonly grouped into statistical learning, shallow machine learning, and deep learning methods. While statistical and shallow models depend heavily on engineered features and data quality, deep learning models can automatically learn hierarchical representations from raw or minimally processed inputs. This capability has made them particularly suitable for complex tasks such as bearing RUL prediction. Despite their advantages, key challenges remain, including the identification of meaningful degradation features and the selection of appropriate models that can effectively learn the relationship between features and RUL [22].

Among deep learning models, Convolutional Neural Networks (CNNs) and Recurrent Neural Networks (RNNs) have been widely adopted. A dual-CNN architecture demonstrated high performance and robustness, although CNNs are typically limited in capturing long-range temporal dependencies due to fixed receptive fields [23]. To address this, RNN variants such as Long Short-Term Memory (LSTM) and Gated Recurrent Units (GRU) have been employed for time-series modeling. For instance, utilized signal enhancement methods including maximum correlation kurtosis deconvolution and multiscale permutation entropy prior to inputting data into an LSTM model optimized via the sparrow search algorithm [24]. Similarly, integrated multi-sensor information within a GRU-based framework to improve prediction performance [25].

Recently, Temporal Convolutional Networks (TCNs) have gained attention for their ability to model long-term temporal patterns through dilated causal convolutions and residual connections. TCNs were applied in conjunction with multi-domain features and segmented lifecycle data, yielding improved performance over conventional time-series models [26]. Transformer-based architectures, known for their efficiency in modeling long sequences, have emerged as promising alternatives. Another study demonstrated strong RUL prediction performance using cumulative-transformed input features within a Transformer framework [27]. Collectively, these developments underscore a growing trend toward leveraging advanced deep learning models for accurate and robust bearing RUL estimation.

Several studies have presented hybrid deep and machine learning architectures, including Transformer-Deep Neural Network (Transformer-DNN) and Decision Tree-Multilayer Perceptron (DT-MLP) models, and digital twin frameworks to improve failure detection, system visualization, and condition monitoring in industrial systems [28,29,30,31]. Recent research has demonstrated the effectiveness of deep learning models in predicting failures in industrial machines. A time-to-failure prediction framework is developed using an enhanced LSTM model trained on real-time sensor data, including current, voltage, and temperature, calibrated through whispering gallery mode optical sensors. The proposed LSTM model achieved high performance with an MAE of 0.83. In comparison, the GRU model attained an MAE of 0.87, CNN achieved 0.93, Deep-MLP recorded 1.44, DNN reached 1.62, Autoencoder (AE) obtained 1.84, and TCN yielded the highest MAE of 2.56. These findings confirm the superior performance of the LSTM model for early failure detection in smart industrial environments [32].

Some studies have investigated unsupervised data augmentation approaches to improve training data quality for RUL prediction in rotating machinery. A Variational Autoencoders-Generative Adversarial Networks (VAE-GAN)-based framework was proposed to generate high-quality time-domain features from bearing vibration signals, aiming to overcome the limitations of real-world data in capturing degradation patterns. This method integrated the generative capabilities of VAE and GAN to synthesize realistic degradation features and employed Dynamic Time Warping (DTW) to calculate similarity-based weights between training and target sequences. The proposed model achieved superior performance on the IMS dataset, particularly for Bearings B2 and B4, yielding RMSE values of 0.041 and 0.045, respectively. These results surpassed those of models trained with original or GAN-only data, highlighting the effectiveness of integrating unsupervised data augmentation with sequence alignment techniques in enhancing RUL prediction performance [33].

Recent studies have demonstrated that integrating the HHO algorithm with LSTM networks significantly improves RUL prediction performance and robustness for energy storage components such as supercapacitors [34]. A hybrid Wild Horse Optimization-based deep learning framework, incorporating attention-based LSTM and Artificial Algae Optimization, has been proposed for feature selection and hyperparameter tuning in short-term load forecasting tasks, demonstrating high prediction performance in smart grid applications [35].

Some studies have investigated advanced deep learning architectures to enhance remaining useful life prediction, including multi-model fusion frameworks and multimodal transfer learning strategies for engines, and deep learning-based approaches for intelligent machinery health management and fault diagnosis [36,37,38]. In addition, recent research has introduced domain knowledge-guided monitoring, deep learning prognostic frameworks, transformer-temporal convolutional feature fusion, and bidirectional sequence modeling with causal discovery for enhanced failure prediction and RUL in complex industrial systems [39,40,41,42].

Several studies have predicted the remaining useful life of rolling bearings using the NASA IMS dataset. SOA-SVM, semi-supervised transfer learning, MLGS-LSTM, and similarity feature fusion with CNNs have all reported competitive normalized MAE and RMSE on IMS2 [43,44,45,46], providing benchmarks for new predictive methods.

Several studies have shown that hybrid ensemble methods improve remaining useful life (RUL) prediction by combining diverse models. Rezazadeh et al. integrated linear regression, k-nearest neighbors (KNN), and Gaussian process regression (GPR) on the NASA C-MAPSS dataset, outperforming individual models [47]. Other ensembles, including decision-tree-based models and stacking approaches, further demonstrate the benefits of hybrid strategies for capturing complex degradation patterns [48,49]. Domain shift, a factor affecting model performance when models trained under specific conditions are applied to different conditions or datasets, has been addressed in structural health monitoring using domain-adaptive graph attention semi-supervised networks for temperature-resilient SHM of composite plates [50]. These findings motivate the transformer-GRU hybrid framework proposed in this study.

The proposed study introduces a novel hybrid HHO–WHO optimization framework integrated with a Transformer-GRU architecture to address critical limitations in existing predictive maintenance methods, including manual hyperparameter tuning, limited generalization across diverse datasets, suboptimal predictive performance, and inadequate temporal modeling of degradation patterns. By leveraging HHO’s global exploration and WHO’s local exploitation, the framework achieves a balanced search of the hyperparameter space, mitigates local optima entrapment, and enhances model generalization. Following hybrid optimization, the Transformer-GRU model achieved an MAE of 0.15 and an RMSE of 0.23 on the TTF dataset, an MAE of 0.01 and an RMSE of 0.02 on the IMS dataset, an MAE of 9.97, an RMSE of 13.56, and score of 254.3 on C-MAPSS FD001, and an MAE of 9.98, an RMSE of 14.57, and score of 320.8 on C-MAPSS FD003. These results demonstrate that the proposed framework not only improves predictive performance but also enhances robustness, scalability, and reliability, outperforming conventional approaches and state-of-the-art baseline models while providing an automated and adaptive solution for complex industrial environments.

## 3. Materials and Methods

### 3.1. Structural Design of Deep Learning Models and Optimization Techniques

A regression-based framework is proposed for failure prediction, incorporating multiple deep learning architectures, including ResNet, Bi-LSTM, Bi-GRU, CNN, DNN, VAE, and a hybrid Transformer-GRU model. ResNet captures hierarchical temporal features via residual connections, whereas Bi-LSTM and Bi-GRU leverage bidirectional recurrent units to model past and future dependencies in sequential data. CNN and DNN focus on spatial feature extraction and nonlinear mapping, while VAE learns latent representations for regression. The Transformer-GRU model combines self-attention mechanisms with GRU layers to capture both contextual relationships and temporal dynamics effectively. To improve predictive performance and generalization, a hybrid HHO–WHO algorithm is employed to tune hyperparameters of the Transformer-GRU and other regression models. The detailed architectures, mathematical formulations, training setups, and optimization strategy are presented in the subsequent subsections.

#### 3.1.1. ResNet Model

ResNets are designed to learn deep hierarchical features by introducing identity shortcut connections that mitigate the degradation problem in deep architectures. In this study, the ResNet architecture for failure prediction comprises stacked residual blocks with one-dimensional convolutional layers, batch normalization, and pooling layers [51,52].

The model begins with a 1D convolutional input layer with 32 filters and a kernel size of 3, employing ReLU activation to extract local temporal features, as shown in Table 1. Batch normalization follows to stabilize training. Two sequential residual blocks are stacked, each consisting of two convolutional layers with 32 filters and kernel size 3, batch normalization, and ReLU activation. Skip connections bypass each residual block, facilitating gradient flow and preserving important input features.

Global average pooling aggregates features across the temporal dimension, reducing the number of parameters. A dropout layer with a rate of 0.01 is applied for regularization. A dense layer with 16 units and ReLU activation refines the extracted features, and a final dense layer with a linear activation function outputs a single scalar value representing the predicted failure time and remaining useful life.

Model hyperparameters prior to optimization are summarized in Table 2. The model uses the Adam optimizer with a learning rate of 0.02 and a batch size of 64. Evaluation is conducted using standard regression metrics, including mean absolute error and root mean squared error.

#### 3.1.2. Bi-LSTM Model

Bi-LSTM networks are designed to capture both past and future temporal dependencies in sequential data. By processing inputs in both forward and backward directions, Bi-LSTM networks are highly effective for regression tasks such as failure prediction [53,54,55]. The architecture combines memory retention, gating mechanisms, and bidirectional learning to improve predictive performance in complex, time-dependent datasets.

The model begins with an input layer for sequential data, feeding into a Bidirectional LSTM layer with 32 hidden units, as presented in Table 3. This bidirectional configuration enables the network to capture both past and future dependencies within the input sequences. The output of the Bi-LSTM layer is passed through a dropout layer with a rate of 0.1 to prevent overfitting by randomly deactivating neurons during training. A fully connected dense layer with 16 units and ReLU activation follows, enhancing nonlinear feature learning. The final dense layer has a single neuron with a linear activation function, outputting the predicted failure time and remaining useful life.

Hyperparameter configurations prior to optimization are summarized in Table 4. The model uses the Adam optimizer with a learning rate of 0.02 and a batch size of 64. The loss function is mean absolute error, and performance is evaluated using MAE and RMSE, providing a comprehensive assessment of predictive performance.

#### 3.1.3. Bi-GRU Model

Bi-GRU networks are a streamlined variant of recurrent neural architectures designed to model temporal dependencies in sequential data. GRUs simplify the internal structure of traditional LSTM units by combining memory mechanisms into fewer gates while still capturing long-term dependencies. By extending GRUs bidirectionally, the model leverages both past and future contexts, making it highly effective for failure prediction tasks in complex systems [56,57,58].

The model begins with an input layer for sequential data, which is fed into a bidirectional GRU layer with 64 hidden units, as detailed in Table 5. This configuration allows the network to capture temporal dependencies in both directions. The GRU output is passed through a dropout layer with a rate of 0.1 to prevent overfitting by randomly deactivating neurons during training. A fully connected dense layer with 32 units and ReLU activation refines the extracted features. The final dense layer consists of a single neuron with a linear activation function, producing the predicted failure time and remaining useful life.

Hyperparameter configurations prior to optimization are summarized in Table 6. The model uses the Adam optimizer with a learning rate of 0.001 and a batch size of 64. Mean absolute error (MAE) is used as the loss function due to its robustness to outliers in regression settings. Performance is evaluated using MAE and root mean squared error (RMSE), providing a comprehensive assessment of predictive performance.

#### 3.1.4. CNN Model

Convolutional neural networks (CNNs) are widely used deep learning architectures designed to automatically extract local features and hierarchical patterns from data. By applying convolutional filters across the input, CNNs efficiently capture correlations between variables while reducing model complexity compared to fully connected networks. In time-series prediction tasks, CNNs are particularly effective at identifying localized temporal dependencies and compressing redundant signals, supporting accurate regression-based forecasting [59,60,61].

The model begins with a one-dimensional convolutional input layer, followed by a Conv1D layer with 16 filters and a kernel size of 5, which extracts local feature patterns from the input, as shown in Table 7. A MaxPooling1D layer with a pool size of 2 reduces the dimensionality of the feature maps while retaining the most salient features. The output is flattened and passed through a fully connected dense layer with 64 neurons and ReLU activation, enabling nonlinear feature mapping. The final dense layer consists of a single neuron with a linear activation function, producing the predicted failure time and remaining useful life.

Hyperparameter configurations prior to optimization are summarized in Table 8. The model uses the Adam optimizer with a learning rate of 0.001 and a batch size of 64. Mean absolute error (MAE) is used as the loss function due to its robustness against outliers in regression tasks. Model performance is evaluated using MAE and root mean squared error (RMSE), providing a comprehensive assessment of predictive performance.

#### 3.1.5. DNN Model

Deep neural networks (DNNs) are feedforward architectures composed of multiple fully connected layers that progressively transform input features into higher-level representations. In regression-based failure prediction tasks, DNNs effectively capture non-linear relationships between operational parameters and the target failure horizon [59,62,63].

The network receives standardized input features through an input dense layer with 64 neurons and ReLU activation, as presented in Table 9. This is followed by two fully connected hidden layers, each with 64 neurons and ReLU activation, enabling the model to learn complex nonlinear feature relationships. After each dense layer, a dropout layer with a rate of 0.05 is applied to prevent overfitting by randomly deactivating neurons during training. The output layer consists of a single neuron with a linear activation function, producing the predicted failure time and remaining useful life.

Hyperparameter configurations prior to optimization are summarized in Table 10. The network uses the Adam optimizer with a learning rate of 0.01 and a batch size of 64. The loss function is mean absolute error (MAE), chosen for its robustness to outliers. Model performance is evaluated using MAE and root mean squared error (RMSE), providing a comprehensive assessment of predictive performance.

#### 3.1.6. VAE Model

Variational autoencoders (VAEs) are generative models that learn compressed latent representations while enforcing smoothness in the latent space through probabilistic constraints. Unlike traditional autoencoders, VAEs estimate a distribution over latent variables using a probabilistic encoder-decoder framework, which promotes generalization and robustness for feature extraction and downstream regression tasks [64,65,66].

The model consists of an encoder, a latent space, a decoder, and a regression network. The encoder includes two fully connected dense layers with 256 and 128 neurons, respectively, each using ReLU activation, as detailed in Table 11. Dropout with a rate of 0.4 is applied to prevent overfitting. The encoder outputs latent features, which are passed to the decoder and a regression network. The decoder mirrors the encoder with two dense layers (128 and 256 neurons) and dropout, reconstructing the input features. The regression network receives the latent embeddings and contains fully connected layers with decreasing neurons (256 to 64) and ReLU activations. Dropout of 0.4 is applied after selected layers, and a final linear neuron produces the predicted failure time and remaining useful life.

Hyperparameters and architecture are summarized in Table 12. The VAE model is optimized using the Adam optimizer with a learning rate of 0.0001 and a batch size of 32. Loss functions include reconstruction loss combined with KL divergence for latent space regularization and MSE for regression. Model performance is evaluated using MAE and RMSE metrics to provide a comprehensive assessment of predictive performance.

#### 3.1.7. Hybrid Transformer-GRU Model

The hybrid Transformer-GRU model integrates the self-attention mechanism of Transformers with the temporal modeling capability of Gated Recurrent Units (GRUs) to capture both spatial correlations and sequential dependencies in failure prediction. The key mathematical formulations of the model are summarized below [56,67,68]. The Transformer block employs attention to model dependencies between features by computing similarity scores between input tokens:

(1) $$Attention\left(Q,K,V\right)=softmax(\frac{QK^{T}}{\sqrt{d_{k}}})V$$

where Q, K, V denote the query, key, and value matrices obtained from linear projections of the input sequence *X*. The factor $\sqrt{d_{k}}$ normalizes the dot product, ensuring stable gradients [67]. This mechanism enables the network to focus on the most relevant features for failure prediction. To capture diverse dependencies, multiple attention heads are used in parallel:

(2) $$MHA\left(X\right)=Concat(h_{1},h_{2},\ldots ,h_{h})W^{O}$$

(3) $$h_{i}=Attention(XW_{i}^{Q},\;\;XW_{i}^{K},\;\;XW_{i}^{V})$$

where each head $h_{i}$ is computed using distinct projection matrices $W_{i}^{Q}$, $W_{i}^{K}$, $W_{i}^{V}$, and $W^{O}$ is a learned output projection. This enhances representation learning by allowing the model to attend to multiple feature relationships simultaneously [67,68]. The GRU layer captures temporal dynamics through gating mechanisms:

(4) $$h_{t}=\left(1-z_{t}\right)*h_{t-1}+z_{t}*{\tilde{h}}_{t}$$

(5) $${\tilde{h}}_{t}=\tanh\left(W_{h}\left[r_{t}*h_{t-1},x_{t}\right]\right)$$

where $h_{t}$ is the hidden state at time step t, $h_{t-1}$ is the previous state, $r_{t}$ and $z_{t}$ are the reset and update gates, and ∗ denotes element-wise multiplication. These gates regulate how much past information is preserved versus how much new information is incorporated [55]. The GRU output is passed through dense layers with dropout and non-linear activation to yield the final prediction:

(6) $$\hat{y}=W_{2}*ReLU\left(W_{1}*Dropout\left(h_{t}\right)+b_{1}\right)+b_{2}$$

where $h_{t}$ is the GRU hidden state, Dropout mitigates overfitting, and $\hat{y}$ represents the predicted failure time and RUL. The integration of Transformer-based attention for feature dependency modeling and GRU-based sequential processing allows the hybrid Transformer-GRU model to effectively exploit both instantaneous sensor features and their temporal evolution, making it well-suited for failure prediction tasks.

**Figure 1:**
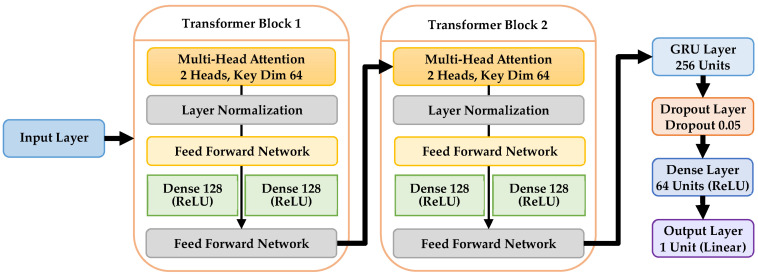
Model architecture of proposed hybrid Transformer-GRU model.

This design places the GRU layer after the Transformer blocks to first extract rich contextual and relational features across input sequences via attention, followed by sequential modeling of temporal dynamics. By processing spatial and inter-feature dependencies through the Transformer before the GRU captures temporal evolution, the model more effectively exploits both instantaneous sensor information and sequential patterns, improving predictive performance compared to alternative arrangements. The structure and components of the Transformer-GRU model are summarized in Table 13. The model begins with an input layer designed for sequential features, followed by two consecutive transformer blocks, as shown in Figure 1. Each block includes a Multi-Head Attention layer with 2 attention heads and a key dimension of 64, followed by layer normalization and a residual connection to retain original input information. A feedforward network composed of two dense layers with 128 neurons each and residual connections follows the attention mechanism, enabling nonlinear feature interactions. After the transformer layers, a single GRU layer with 256 units captures temporal dependencies across input features. A dropout layer with a rate of 0.05 is applied for regularization. The GRU output is then passed through a dense layer with 64 neurons and ReLU activation, and finally a linear output neuron predicts the continuous target variable (RUL and failure time).

The training configuration and evaluation measures for the Transformer-GRU model are outlined in Table 14. The model is trained using the Adam optimizer with a learning rate of 0.001 and a batch size of 64. The MAE loss function is used, providing robustness to outliers. Predictive performance is assessed using multiple metrics including MAE, and RMSE, which together offer a comprehensive performance of performance.

#### 3.1.8. Hybrid HHO–WHO Algorithm

This approach utilizes a hybrid optimization strategy that combines HHO and WHO to efficiently tune hyperparameters for the Transformer-GRU model and other regression models. The hybrid framework alternates between HHO and WHO updates to maintain a balance between global exploration and local exploitation. The key mathematical formulations underlying the optimizer are presented below [69,70]. To initiate the dynamic behavior of the Harris Hawks optimizer, the escaping energy of the prey is computed as follows:

(7) $$E=2*E_{0}\cdot (1-\frac{t}{T})$$

where E represents the escaping energy, $E_{0}$ is a constant that controls the initial energy level (set to 2 in the implementation), t denotes the current generation number, and T is the maximum number of generations. The escaping energy decreases linearly over time, allowing the optimizer to shift from exploration to exploitation as the iterations progress. When the absolute value of the escaping energy $\left|E\right|$ is greater than or equal to 1, the algorithm enters the exploration phase. In this phase, the hawk’s position is updated using the following rule:

(8) $$X_{t+1}=\left\{\begin{array}{c} X_{rand}-r_{1}\cdot \;\left|X_{rand}-2r_{2}\cdot X_{t}\right|\;\;,\;\;\;\;if\;q<0.5\\ \left(X_{best}-\bar{X}\right)-r_{3}\cdot △,\;\;\;\;\;\;\;\;\;\;\;\;\;\;\;\;\;\;\;\;\;otherwise \end{array}\right.$$

where $X_{t}$ is the current position of the candidate hawk, $X_{rand}$ is a randomly selected position from the population, and $X_{best}$ represents the best solution found so far. $\bar{X}$ is the average position of the current population, while $r_{1}$, $r_{2}$, $r_{3}$ ***ϵ*** (0, 1) are random scalars that control the step size. The vector △ represents a random perturbation. This formulation enables the optimizer to explore diverse regions of the search space by introducing stochastic jumps. If the escaping energy falls below the threshold $\left|E\right|\;$< 1, the optimizer transitions into the exploitation phase. During this phase, the hawk’s position is updated as follows:

(9) $$X_{t+1}=\left\{\begin{array}{c} X_{best}-E\cdot \;\left|X_{best}-X_{t}\right|\;\;,\;\;\;\;\;\;\;\;\;\;\;\;\;\;\;if\;r\geq 0.5\\ \left(X_{best}-E\right)\cdot \left|J\cdot X_{best}-X_{t}\right|,\;\;\;\;\;\;\;\;\;otherwise \end{array}\right.$$

where $X_{best}$ is the current global best position, $X_{t}$ is the current hawk position, E is the previously defined escaping energy, and J=2(1−rand()) is a coefficient that introduces adaptive step scaling. r ***ϵ*** (0, 1) is a random number used to stochastically select between the two exploitation strategies. This equation refines the hawk’s position by drawing it closer to the global optimum, thereby improving convergence. To complement the behavior of HHO and enhance convergence speed and stability, the Wild Horse Optimization update is alternated with the HHO update. The WHO-based position update is given by:

(10) $$X_{t+1}=X_{best}-A\cdot \left|C\cdot X_{best}-X_{t}\right|$$

where $A=2ar_{1}$ and $C=2r_{2}$ are acceleration and herd influence coefficients, respectively. The variable a=2(1−rand())  introduces dynamic adaptation in movement intensity, while $r_{1}$ and $r_{2}$ are random values in the range (0, 1). $X_{best}$ is the global best solution, and $X_{t}$ is the current candidate position. This rule simulates herd behavior by guiding each solution toward the best candidate while preserving diversity and preventing premature convergence.

### 3.2. Procedural Architecture of the Proposed Hybrid HHO–WHO Hyperparameter Optimization Framework

**Figure 2:**
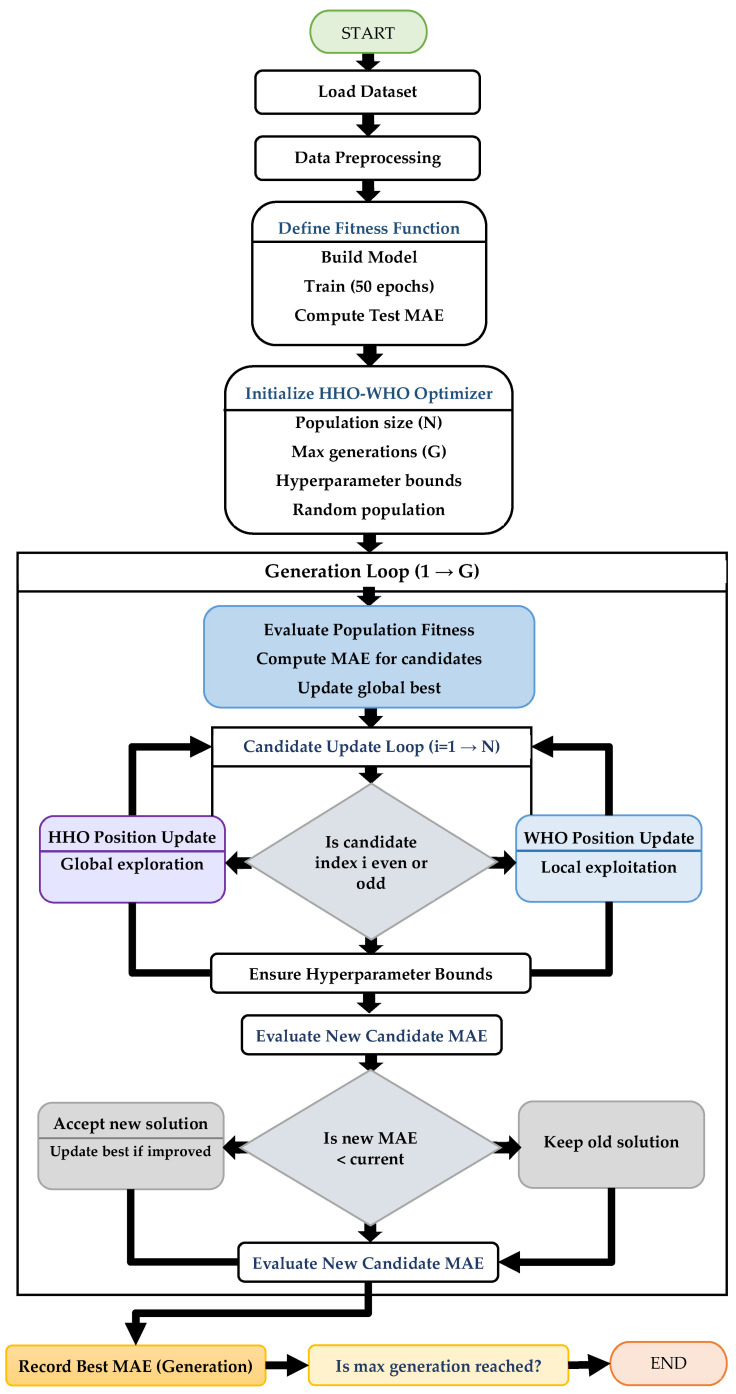
Procedural architecture of the proposed hybrid HHO–WHO hyperparameter optimization framework for Transformer-GRU model.

The flowchart illustrated in Figure 2 presents the procedural architecture of the proposed hybrid Harris Hawks Optimization–Wild Horse Optimization (HHO–WHO) framework for hyperparameter tuning of a Transformer-GRU-based deep learning regression model. Unlike conventional optimization pipelines, the proposed framework integrates metaheuristic search directly with iterative deep model training and testing, aiming to minimize the prediction error measured using the mean absolute error (MAE). The framework is specifically designed for sensor-driven failure prediction, utilizing operational and system-level input features.

The process begins with loading the sensor dataset, followed by data preprocessing, including train-test splitting and feature standardization. Standardization is applied to ensure numerical stability and to improve convergence behavior during deep model training, as reflected in the preprocessing stage of the flowchart. Subsequently, an initial population of candidate solutions is randomly generated within predefined hyperparameter bounds, consistent with the initialization phase shown in Figure 2. Each candidate encodes a unique combination of hyperparameters, including learning rate, number of GRUs, number of dense units, dropout rate, number of attention heads, number of GRU layers, and number of dense layers, all of which directly influence the representational capacity and generalization performance of the Transformer-GRU model.

The optimization process proceeds iteratively over a fixed number of generations. At the beginning of each generation, the fitness of all candidates is evaluated by constructing a Transformer-GRU model using the candidate’s hyperparameters, training it for a fixed number of epochs, and computing the MAE on a held-out test set. This explicit model-based evaluation strategy, highlighted in the (Evaluate Population Fitness) block of the flowchart, ensures that hyperparameter selection is guided by true predictive performance rather than surrogate approximations.

Following fitness evaluation, the algorithm enters the candidate update loop, where a hybrid position update mechanism is applied based on candidate index parity, as depicted in the decision node of the flowchart. Candidates with even indices are updated using the Harris Hawks Optimization (HHO) strategy, which emphasizes global exploration, while candidates with odd indices are updated using the Wild Horse Optimization (WHO) strategy, focusing on local exploitation. This alternating update scheme is a key enhancement of the proposed framework, enabling an effective balance between exploration and exploitation within the hyperparameter search space. After each update, boundary constraints are enforced to ensure feasibility of the updated hyperparameters, and the new candidate solution is re-evaluated using MAE. If the updated candidate yields a lower MAE than its previous state, the solution is accepted and the global best is updated accordingly; otherwise, the previous solution is retained, as illustrated in the acceptance-rejection decision blocks. The best MAE value is recorded at the end of each generation, providing a clear convergence trace across iterations.

Upon reaching the maximum number of generations, the algorithm terminates and outputs the globally optimal hyperparameter configuration corresponding to the minimum MAE observed during the optimization process. The proposed HHO–WHO framework, as summarized in the flowchart, offers a systematic and reproducible approach that tightly couples metaheuristic optimization with deep learning model evaluation, making it particularly suitable for predictive maintenance and failure estimation tasks in industrial systems.

### 3.3. Predicting Modeling

The hyperparameter optimization ranges for all evaluated models using the hybrid HHO–WHO algorithm is summarized in Table 15. This hybrid optimization framework integrates the exploration capabilities of Harris Hawks Optimization with the exploitation strengths of the Wild Horse Optimization Algorithm to efficiently identify optimal configurations for each model, enabling robust predictive performance across diverse tasks. In addition to the hyperparameter ranges, hyperparameters were encoded as Continuous or Discrete variables based on their nature. Learning rates and dropout rates are Continuous, allowing fine-grained optimization, while units, layers, filters, kernel sizes, and attention heads are Discrete, represented as integer values. This encoding ensures that the hybrid HHO–WHO algorithm can properly handle both types of hyperparameters during exploration and exploitation. This choice is based on each parameter’s role: Continuous parameters allow smooth gradient-based updates for precise training control, while Discrete parameters define integer-valued architectural elements to maintain valid network structures.

For the ResNet model, a deep convolutional architecture that leverages residual connections to facilitate gradient flow, four hyperparameters were optimized. The learning rate is Continuous and varied from 1 × 10^−5^ to 1 × 10^−2^. This allows smooth adjustment of gradient updates during training. The number of convolutional filters is Discrete and optimized between 16 and 128. Filters are integer-valued and define the feature extraction capacity. Dense layer units are Discrete and tuned from 16 to 128. Integer units determine network expressiveness. Dropout rate is Continuous and explored within 0.0 to 0.5. Continuous dropout enables flexible regularization.

The Bi-LSTM model, which processes sequences in both forward and backward directions to capture temporal dependencies, had its learning rate Continuous in the range 1 × 10^−5^ to 1 × 10^−2^. Continuous learning rate allows precise gradient updates for sequence learning. The number of LSTM units and dense units are Discrete, both optimized between 16 and 128. Units are integer values controlling memory and representation capacity. Dropout rate is Continuous with values from 0.0 to 0.5. Dropout rate is continuous to fine-tune regularization strength. Similarly, the Bi-GRU model, a simplified variant of LSTM with fewer parameters, employed the same optimization ranges and encoding for learning rate, GRUs, dense units, and dropout rate.

For the CNN model, four hyperparameters were tuned. Learning rate is Continuous, set between 1 × 10^−5^ and 0.1. This allows precise control of training updates. Convolutional filters are Discrete, optimized from 8 to 128. Filters are integer-valued to define feature maps. Kernel size is Discrete, ranging from 2 to 5. Kernel size must be an integer representing receptive field. Dense units are Discrete, varied from 16 to 256. Units are integers to define layer capacity.

The DNN model, consisting of fully connected layers, had its learning rate Continuous, optimized from 1 × 10^−5^ to 0.1. Continuous learning rate allows smooth optimization. Dense units are Discrete, explored between 16 and 256. Integer units define layer capacity. Dropout rate is Continuous, ranging from 0.0 to 0.5. Allows flexible regularization. The number of dense layers is Discrete, tuned from 1 to 4. Layers are integer counts, defining network depth.

The VAE model, designed for probabilistic latent space representation, optimized three hyperparameters: learning rate Continuous from 1 × 10^−4^ to 5 × 10^−3^. Continuous learning rate allows precise updates in stochastic training. Hidden units Discrete between 16 and 2048. Latent space dimension is integer-valued. Dropout rate Continuous from 0.0 to 0.3. Allows fine control of regularization.

The Transformer-GRU model, combining the self-attention mechanism of Transformers with GRU sequential processing, involved seven hyperparameters. Learning rate is Continuous. Precise control of optimization steps. GRUs and dense units are Discrete. Integer values define neurons for memory and representation. Dropout rate is Continuous. Flexible regularization. Attention heads, GRU layers, and dense layers are Discrete. Integer counts define network structure and parallel attention mechanisms.

These comprehensive optimization ranges and discrete vs. continuous encodings ensure that the hybrid HHO–WHO algorithm can thoroughly explore and exploit the hyperparameter space, identifying optimal configurations to achieve superior predictive performance across varied datasets and modeling tasks.

### 3.4. TTF Dataset Development

**Figure 3:**
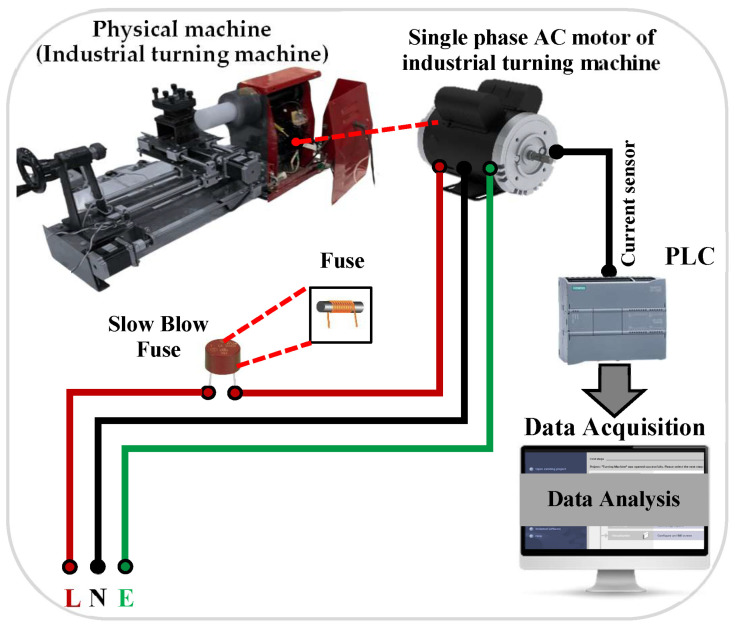
TTF dataset setup.

An advanced data acquisition system was employed to collect the failure time dataset of the industrial turning machine, as illustrated in Figure 3. This system integrates sensors and a control framework that monitors machine performance and prevents failures [32]. The central element is a programmable logic controller (PLC) S7-1200 (Siemens AG, Munich, Germany), which communicates with the HMI and the totally integrated automation portal (TIA Portal v16) software over TCP/IP. Data streams from four main input features, including current, operating system, velocity, and operating time are collected for modeling purposes, while AC voltage and temperature sensors are used solely for motor protection and anomaly detection. Additionally, an overload fuse is incorporated to protect the motor and ensure reliable operation while assembling a comprehensive dataset for analysis. The failure time of the machine is analyzed in relation to the overload currents of the AC motor, which vary with different cutting tool speeds. The dataset, consisting of 18,567 records, is utilized to train machine and deep learning models for predicting failures accurately, thereby improving machine dependability and prolonging operational duration. Each record in the dataset represents a one-second interval, providing a uniform sampling frequency for the analysis. Table 16 presents a statistical analysis of the failure time dataset used for modeling. The table reports the total number of samples, the number of input features employed in the predictive models, and the output variable (failure time). Additionally, descriptive statistics of the output variable, including minimum, maximum, mean, and standard deviation are provided to characterize the distribution of the data. Each record corresponds to a one-second interval, indicating a sampling frequency of 1 Hz across the dataset.

### 3.5. IMS Dataset Development

This is an accelerated life test dataset of bearings, which is provided by the Center for Intelligent Maintenance Systems (IMS) at the University of Cincinnati [33]. Four test bearings were mounted on one shaft driven by an AC motor and coupled by rub belts. The rotational speed was kept constant at 2000 rpm. In this experiment, Rexnord ZA-2115 double-row bearings (Rexnord Technical Services, Milwaukee, WI, USA) were tested. A radial load of 6000 lbs was added to the shaft and bearings by a spring mechanism. The data were collected every 10 min while the bearings were rotating. The sample rate was 20 kHz, and the data length was 20,480 points. Three sets of tests were carried out using the experimental setup provided in the IMS dataset.

In this study, the IMS bearing dataset is organized into multiple experimental test runs, such as the second test (2nd_test), each comprising a temporally ordered sequence of vibration signal recordings. Each file encapsulates 20,480 data points sampled at a rate of 20 kHz, with recordings collected approximately every 10 min. The dataset from the second test (2nd_test) integrates vibration data from all four bearings (B1, B2, B3, and B4) into a unified dataset, which is subsequently used for both training and testing purposes.

This structured time-series arrangement enables detailed examination of progressive bearing degradation under continuous operation. This dataset provides a foundation for time-series-based prognostic modeling, particularly for estimating the remaining useful life of rotating machinery components under continuous degradation. Table 17 summarizes the statistical characteristics of the IMS bearing dataset after computing the RUL for all four bearings of second test (2nd_test). The dataset comprises a total of 3936 samples with 48 extracted features, including time-domain metrics such as mean, standard deviation, skewness, kurtosis, entropy, RMS, peak-to-peak, crest factor, clearance, shape factor, impulse factor, and maximum value, calculated for each bearing. The output variable, RUL, exhibits a minimum value of 1150 cycles and a maximum of 2192 cycles, with a mean of 1667.75 cycles and a standard deviation of 284.88 cycles. These statistics indicate a sufficiently diverse distribution of the RUL, which can support robust modeling and evaluation in predictive maintenance applications, such as Transformer-GRU based prognostics.

### 3.6. C-MAPSS Dataset Development

Collecting full-lifecycle monitoring data from aero-engines in real-world conditions is inherently challenging due to the prolonged duration of degradation phenomena. The operational span from initial healthy conditions to complete system failure often extends over several months or even years, making comprehensive experimental data acquisition extremely time-consuming and resource-intensive. This difficulty is further exacerbated by strict industrial safety regulations, which prohibit in-service failures. Any unexpected malfunction or damage could potentially trigger catastrophic failure cascades, thereby limiting the availability of complete degradation datasets under actual operational environments [71]. Consequently, the scarcity of data representing terminal degradation stages imposes significant constraints on the validation of prognostic models, necessitating the use of controlled simulations or accelerated degradation experiments. Given the complexities of gathering extensive real-world engine data, most contemporary research relies on datasets generated from high-fidelity accelerated degradation testbeds rather than in-service machinery. The NASA Prognostics Center of Excellence (PCoE) has addressed this limitation by providing a comprehensive Prognostics Data Repository, from which the C-MAPSS dataset, utilized in this study, is presented. The C-MAPSS dataset comprises multiple multivariate time-series records, each corresponding to a different engine, effectively representing a fleet of engines of the same design type. Each engine exhibits varying degrees of initial wear and manufacturing variability, which are considered normal operating conditions rather than fault occurrences and are not disclosed to the user. The dataset includes three operational parameters that significantly influence engine behavior, alongside measurements from numerous sensors. Additionally, all signals incorporate realistic sensor noise to emulate actual operational conditions [72]. The C-MAPSS dataset is constructed using a sophisticated gas turbine performance simulation model, capturing the progressive degradation of key rotating components, including the fan, low-pressure compressor (LPC), high-pressure compressor (HPC), high-pressure turbine (HPT), and low-pressure turbine (LPT). The dataset is divided into four subsets (FD001–FD004), each reflecting different operational complexities and fault modes. Taking FD001 as an example, it contains complete operational cycle records for 100 engines, including both training and test sets, along with corresponding true RUL values. In the training set, degradation gradually increases until total system failure, while the test set comprises truncated sequences that end before failure occurs. The primary objective in predictive modeling using C-MAPSS is to construct regression models capable of estimating the RUL. This task involves predicting, based on the truncated time-series data in the test set, the number of additional cycles each engine can operate beyond its last observed cycle until functional failure. Model performance is evaluated by comparing predicted RUL values against the actual remaining cycles. The data sampling rate for the FD001 and FD003 subsets corresponds to one record per engine operating cycle, providing a detailed temporal resolution of engine behavior across its lifecycle.

The FD001 subset of the C-MAPSS dataset comprises 20,631 samples, each described by 25 features: three operational settings and 22 sensor measurements. The output variable is the RUL, representing the number of operational cycles remaining until failure. RUL values range from 0 to 125 cycles, with a mean of 86.83 and a standard deviation of 41.67, reflecting gradual engine degradation across units. These characteristics provide a robust basis for training and evaluating predictive models for engine prognostics. The dataset statistics are summarized in Table 18.

The FD003 subset consists of 24,720 samples, each with 25 features encompassing three operational settings and 22 sensor measurements. The RUL output spans from 0 to 125 cycles, with a mean of 93.14 and a standard deviation of 40.63, reflecting progressive degradation under more complex operational conditions compared to FD001. These statistics provide essential information for model development and evaluation. The dataset statistics are summarized in Table 19.

### 3.7. Data Preprocessing

The TTF dataset, comprising operational features, was preprocessed prior to model development. The target variable was defined as the time-to-failure, and the dataset was partitioned into training (80%) and test (20%) subsets using a fixed random seed to ensure reproducibility. To address feature scale variations and enhance numerical stability, all input variables were standardized using z-score normalization. The processed data were then reshaped into a three-dimensional format to ensure compatibility with sequential deep learning architectures. This preprocessing pipeline ensured consistent feature representation and reliable input structure for subsequent model training.

The IMS bearing dataset was first processed by segmenting raw vibration signals collected from multiple sensor channels. For each segment, a set of time-domain statistical features was extracted, including mean, standard deviation, skewness, kurtosis, entropy, root mean square (RMS), peak-to-peak value, crest factor, clearance factor, shape factor, and impulse factor. These features were computed for all available bearing channels to capture diverse signal characteristics. Subsequently, the RUL labels were constructed by defining failure cycles for each bearing and computing the difference between the current operating cycle and the corresponding failure threshold. Afterward, missing values and infinite values were handled through forward filling and data cleansing to ensure numerical stability. To reduce scale disparities across features, a min-max normalization was applied to both the extracted features and RUL targets, scaled to the range from 0 to 0.95, ensuring consistent input magnitude. The dataset was structured into sequential input windows of fixed length to preserve temporal dependencies, and then partitioned into training and test subsets using an 80/20 split with stratified sampling, ensuring that the distribution of RUL values was consistently represented across both sets.

The C-MAPSS datasets FD001 and FD003 were processed using an identical pipeline to ensure consistency across different operational conditions and facilitate comparative evaluation. Initially, engine run-to-failure trajectories were loaded, where each record included operational settings and multiple sensor measurements. The remaining useful life for each engine unit was computed by subtracting the current cycle from the last observed cycle for that unit, with values capped at a maximum threshold of 125 cycles to limit the influence of extreme values, following standard practice in C-MAPSS studies. To enhance the discriminative capability of the input space, domain-informed features were engineered, including heat-related indices such as the mean of temperature sensors, pressure differentials, rotational speed deviations, and smoothed sensor readings derived via rolling-window operations. Constant and non-informative features were identified and removed to improve model learning efficiency. Given the variations in operating conditions across engines, a condition-specific normalization strategy was applied using RobustScaler.

The temporal structure of the dataset was preserved by constructing fixed-length sliding windows of consecutive cycles with a window size of 50 cycles, selected empirically based on engine degradation patterns, with each window associated with the remaining useful life of its last cycle. The dataset was then partitioned into training and test subsets using an 80/20 split at the engine level, ensuring no information leakage across engines and supporting robust model generalization. These preprocessing steps, including feature engineering, outlier removal using Local Outlier Factor with a contamination level of 0.05, scaling, and windowing, were applied identically to both FD001 and FD003, ensuring reproducibility and comparability of model performance across datasets.

### 3.8. Performance Evaluation Using MAE, RMSE, Score, and Cross-Validation

The model’s performance was evaluated using three metrics: Mean Absolute Error (MAE), Root Mean Squared Error (RMSE), and the NASA Score for C-MAPSS datasets [32]. These metrics were computed on the standard train-validation split to assess baseline predictive performance. To ensure robustness and mitigate overfitting or data leakage, MAE, RMSE, and Score were further evaluated using 5-fold cross-validation. MAE quantifies the average magnitude of prediction errors as the absolute differences between predicted and actual values:

(11) $$MAE=\frac{1}{n}\;.\;\sum_{i=1}^{n}\left|y_{i}-{\hat{y}}_{i}\right|$$

where n represents the total number of samples, $y_{i}$ is the actual value, and ${\hat{y}}_{i}$ denotes the predicted value. RMSE measures the square root of the average squared differences between predicted and actual values:

(12) $$RMSE=\sqrt{\frac{1}{n}\;.\;\sum_{i=1}^{n}{\left(y_{i}-\;{\hat{y}}_{i}\right)}^{2}\;}$$

where n represents the total number of samples, $y_{i}$ is the actual value, and ${\hat{y}}_{i}$ is the predicted value.

5-Fold Cross-Validation (5-CV) divides the dataset into five equal folds to evaluate model robustness. In each iteration, four folds are used for training and the remaining fold for validation. The final performance is the average across all folds:

(13) $$CV\;Metric=\frac{1}{k}\sum_{j=1}^{k}{Metric}_{j},\;\;\;\;\;\;\;\;\;\;\;k=5$$

where k is the number of folds, ${Metric}_{j}$ represents the value of the metric (MAE, RMSE) for the $j^{th}$ fold, and n in each fold represents the number of samples in that fold. For the C-MAPSS datasets, the NASA Score quantifies prediction performance with asymmetric penalties for late predictions:

(14) $$S_{i}=\left\{\begin{array}{c} e^{{-E}_{i}/10}-1,\;\;\;\;\;\;\;\;\;\;\;\;\;\;\;E_{i<0}\\ e^{{-E}_{i}/13}-1,\;\;\;\;\;\;\;\;\;\;\;\;\;\;\;E_{i\geq 0} \end{array}\right.$$

(15) $$Score=\sum_{i=1}^{N}S_{i\;\;}$$

where $E_{i}={\hat{y}}_{i}-y_{i}$ is the prediction error for the *i*-th sample, ${\hat{y}}_{i}$ is the predicted RUL, $y_{i}$ is the true RUL, and *N* is the total number of test samples. This asymmetric formulation penalizes late predictions more heavily than early predictions, reflecting the operational risk of delayed maintenance.

Collectively, MAE, RMSE, and NASA Score provide a rigorous and standardized evaluation of the Transformer-GRU model’s predictive performance. All three metrics were computed not only on the standard train-test split but also using 5-fold cross-validation to ensure robustness and mitigate overfitting or data leakage across datasets. This approach guarantees that the reported performance reflects consistent and reliable predictive capability.

## 4. Experimentation and Analysis of Results

### 4.1. Model Experiment Results

#### 4.1.1. ResNet Model Parameters Optimization

The optimal hyperparameters for the ResNet model were identified through the application of a hybrid metaheuristic optimization approach combining HHO and WHO algorithms. As shown in Table 20, the resulting configuration yielded a learning rate of 0.0015, 41 filters in the convolutional layers, 19 units in the fully connected dense layer, and a dropout rate of 0.03. The optimized hyperparameter set contributed to enhanced model convergence and improved performance, demonstrating the effectiveness of the HHO–WHO technique in fine-tuning deep learning models for failure detection tasks.

#### 4.1.2. BI-LSTM Model Parameters Optimization

The BI-LSTM model was optimized using the hybrid HHO–WHO algorithm to determine the most effective set of hyperparameters. As presented in Table 21, The optimization process yielded a learning rate of 0.0053, 59 units in the bidirectional LSTM layers, 26 units in the dense layer, and a dropout rate of 0.01. These hyperparameter settings enabled improved generalization and stability during training, indicating that the HHO–WHO algorithm effectively tailored the BI-LSTM model for enhanced performance in the context of failure detection.

#### 4.1.3. BI-GRU Model Parameters Optimization

To enhance the performance of the BI-GRU model, a hybrid HHO–WHO technique strategy was employed to fine-tune its hyperparameters. As detailed in Table 22, the optimal configuration obtained includes a learning rate of 0.0024, 44 GRUs in the bidirectional layer, 16 units in the dense output layer, and no dropout regularization (dropout rate of 0.0). This tailored hyperparameter set contributed to efficient model training and reliable detection outcomes, confirming the suitability of the HHO–WHO approach for optimizing recurrent architectures in failure detection tasks.

#### 4.1.4. CNN Model Parameters Optimization

The CNN model was optimized using the hybrid HHO–WHO algorithm to identify the most effective hyperparameter configuration. As shown in Table 23, the optimization process resulted in a learning rate of 0.0019, 37 convolutional filters, a kernel size of 3, and 188 units in the dense layer. These settings allowed the CNN to extract meaningful hierarchical features from the input data while maintaining efficient learning dynamics. The dropout rate was not incorporated in the final model, suggesting that the model architecture was sufficiently robust without additional regularization. The results indicate that the HHO–WHO algorithm successfully tuned the CNN model for accurate predictive performance.

#### 4.1.5. DNN Model Parameters Optimization

The DNN model was optimized using the hybrid HHO–WHO algorithm to determine the best hyperparameters set. Table 24 summarizes the results, showing a learning rate of 0.0044, 80 units in the dense layer, a single dense layer, and a dropout rate of 0.0. These optimized hyperparameters ensured efficient training and strong representation capacity while avoiding overfitting, highlighting the effectiveness of the HHO–WHO approach in identifying the most suitable configuration for deep feedforward architectures.

#### 4.1.6. VAE Model Parameters Optimization

The VAE model was optimized using the hybrid HHO–WHO algorithm to select the optimal hyperparameters for effective latent space representation and reconstruction. Table 25 presents the results, with 1024 hidden units, a dropout rate of 0.1, and a learning rate of 0.001. These hyperparameters allowed the VAE to learn a rich latent encoding while maintaining stable training. The hybrid HHO–WHO algorithm efficiently explored the parameter space, enabling the selection of hyperparameters that enhance model performance in generative and predictive tasks.

#### 4.1.7. Transformer GRU Model Parameters Optimization

The Transformer-GRU model, which integrates the attention mechanism of the Transformer with sequential processing of the GRU, was optimized using the hybrid HHO–WHO algorithm. As detailed in Table 26, the selected hyperparameters include a learning rate of 0.00094, 44 GRUs, 16 dense units, dropout rate of 0.0, 3 attention heads, 2 GRU layers, and 1 dense layer. These optimized values ensure that the model captures sequential dependencies effectively while leveraging attention mechanisms for enhanced contextual feature learning. The results demonstrate that HHO–WHO is capable of finely tuning hybrid models to achieve superior predictive performance.

### 4.2. Performance of Predictive Models of TTF Dataset

The TTF dataset serves as a benchmark for evaluating the predictive performance of different deep learning models in time-to-failure estimation. Table 27 presents the performance of seven models, comparing their prediction errors before and after optimization.

As shown in Table 27, prior to optimization, Transformer-GRU achieves the highest predictive performance, with a Test MAE of 0.718 and a Test RMSE of 1.313, indicating its superior capability in capturing temporal dependencies. CNN also performs well, achieving a Test MAE of 1.616 and a Test RMSE of 3.381. Bi-GRU and Bi-LSTM provide competitive results, with Test MAE values of 2.031 and 2.945, and Test RMSE values of 2.788 and 4.528, respectively. DNN and VAE show moderate performance, with Test MAE of 2.177 and 3.610, and Test RMSE of 3.929 and 4.918. ResNet exhibits the least accurate predictions, with a Test MAE of 4.484 and a Test RMSE of 5.486.

After applying optimization, all models demonstrate notable improvements in predictive performance. Transformer-GRU maintains the best performance, with an Optimized Test MAE of 0.152 and an Optimized Test RMSE of 0.232, approaching near-perfect predictions. CNN and VAE also show substantial improvements, achieving Optimized Test MAE of 0.672 and 0.821, and Optimized Test RMSE of 1.292 and 1.371, respectively. Bi-GRU and Bi-LSTM offer competitive optimized results, with Optimized Test MAE of 1.026 and 1.325, and Optimized Test RMSE of 1.491 and 1.944. DNN shows moderate gains, with an Optimized Test MAE of 1.839 and an Optimized Test RMSE of 3.663. ResNet, while still the least accurate, improves significantly, recording an Optimized Test MAE of 1.583 and an Optimized Test RMSE of 2.738.

**Figure 4:**
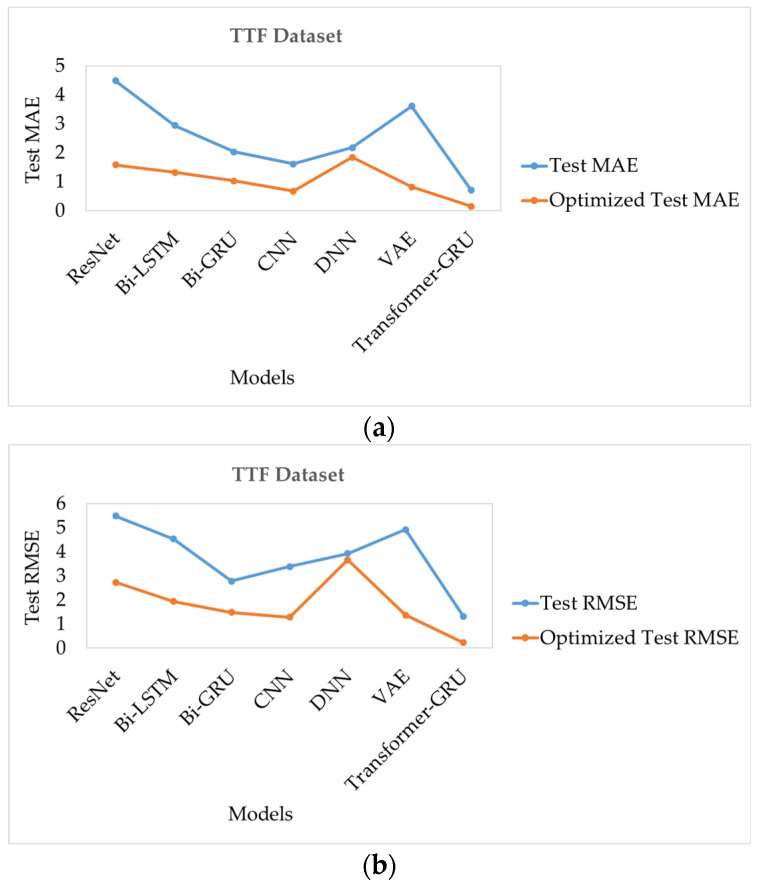
Error Performance Curves of different models on the TTF dataset before and after optimization: (**a**) MAE, (**b**) RMSE.

These results demonstrate that optimization significantly enhances the predictive performance of all models on the TTF dataset, with Transformer-GRU consistently outperforming other architectures, as presented in Figure 4a,b.

**Figure 5:**
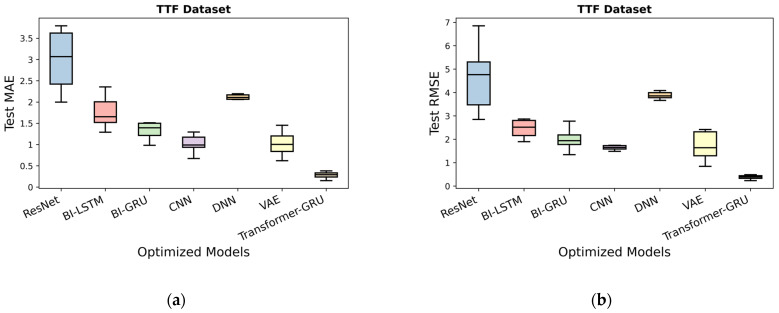
Distribution of optimized test errors for different models on the TTF dataset: (**a**) MAE, (**b**) RMSE.

The optimized predictive performance of the evaluated models on the TTF dataset is analyzed through the distribution of test MAE values. Transformer-GRU achieves the lowest median MAE with a compact interquartile range, indicating high prediction performance and strong stability across test cases. CNN and VAE demonstrate relatively low errors, although with moderately higher dispersion. Bi-GRU, Bi-LSTM, DNN, and ResNet exhibit higher median MAE values and wider distributions, reflecting reduced stability and robustness under optimized conditions, as illustrated in Figure 5a.

The optimized test RMSE distributions show similar patterns. Transformer-GRU records the lowest RMSE values with minimal dispersion, confirming stable performance and effective limitation of large prediction deviations. CNN and VAE maintain competitive performance with moderate variability, whereas Bi-GRU and Bi-LSTM show increased dispersion. ResNet and DNN present the highest RMSE values and the broadest spreads, indicating greater sensitivity to extreme prediction errors. These findings are consistent with the MAE analysis and are clearly presented in Figure 5b. Transformer-GRU demonstrates superior predictive performance and stability compared to all other evaluated models, highlighting its robustness for TTF estimation tasks.

### 4.3. Performance of Predictive Models of IMS Dataset

The predictive performance of different models on the IMS dataset was evaluated before and after optimization, as summarized in Table 28. Optimization consistently improves the performance of all models, demonstrating the effectiveness of the applied strategy in enhancing model predictions.

For MAE, Transformer-GRU achieves the lowest error, decreasing from 0.0356 before optimization to 0.0126 after optimization, indicating the highest predictive performance. ResNet’s MAE reduces from 0.0836 to 0.0267, Bi-LSTM from 0.1149 to 0.0819, Bi-GRU from 0.0867 to 0.0565, CNN from 0.1011 to 0.0834, DNN from 0.1037 to 0.0926, and VAE from 0.1611 to 0.1487. These reductions highlight the overall improvement in model precision following optimization.

**Figure 6:**
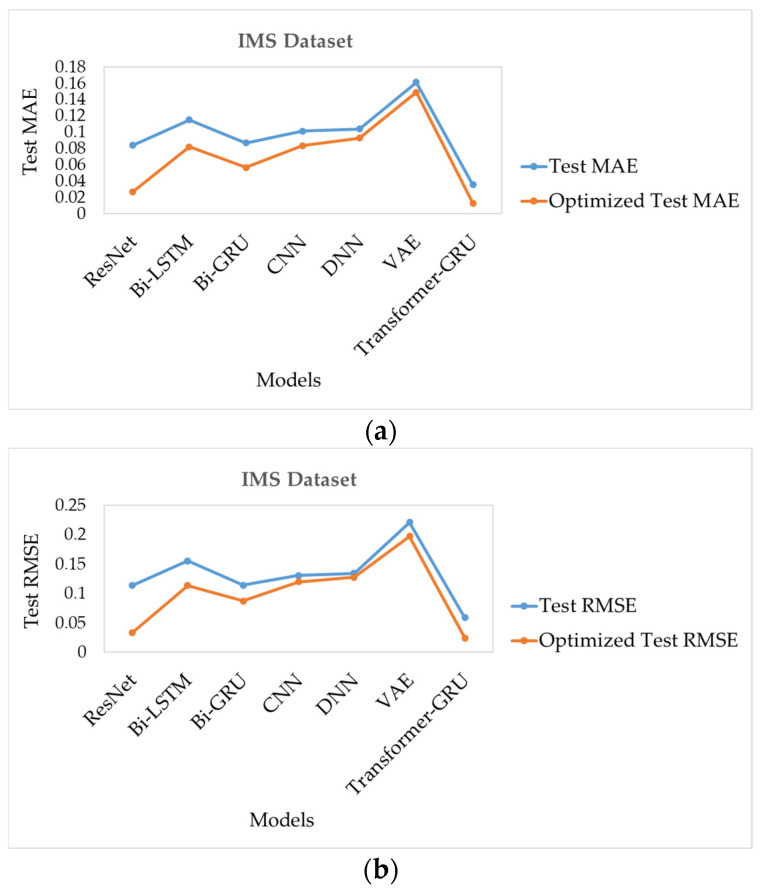
Error Performance Curves of different models on the IMS dataset before and after optimization: (**a**) MAE, (**b**) RMSE.

For RMSE, Transformer-GRU again demonstrates superior performance, with values dropping from 0.0586 to 0.0242. ResNet’s RMSE decreases from 0.1134 to 0.0328, Bi-LSTM from 0.1556 to 0.1134, Bi-GRU from 0.1143 to 0.0871, CNN from 0.1305 to 0.1198, DNN from 0.1340 to 0.1273, and VAE from 0.2211 to 0.1974. These results confirm that Transformer-GRU consistently outperforms all other models in both MAE and RMSE, as illustrated in Figure 6a,b.

**Figure 7:**
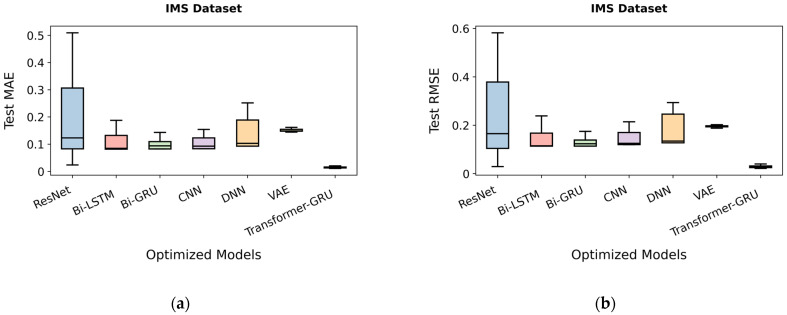
Distribution of optimized test errors for different models on the IMS dataset: (**a**) MAE, (**b**) RMSE.

Test MAE illustrates the comparative prediction errors of the evaluated models on the IMS dataset. ResNet and DNN exhibit relatively high median MAE values and wider interquartile ranges, indicating less accurate and less consistent predictions. Bi-LSTM, Bi-GRU, CNN, and VAE show lower median errors and narrower distributions, reflecting improved predictive performance and stability, as shown in Figure 7a. Transformer-GRU demonstrates the lowest median MAE with minimal variation, indicating superior precision and robustness across the test samples.

Similarly, RMSE quantifies the squared deviations of predictions and confirms these performance patterns in model behavior. ResNet and DNN again show higher median RMSE and greater spread, suggesting susceptibility to larger prediction errors. Bi-LSTM, Bi-GRU, CNN, and VAE achieve moderate RMSE reductions, while Transformer-GRU consistently attains the lowest RMSE values with tightly clustered results, as shown in Figure 7b. These observations underscore the superior performance and stability of Transformer-GRU in accurately modeling and predicting the IMS dataset.

### 4.4. Performance of Predictive Models of C-MAPSS FD001 Dataset

Accurate prediction of remaining useful life is essential for the effective maintenance and reliability of engineering systems. Different deep learning models exhibit varying levels of predictive performance on the C-MAPSS FD001 dataset, as summarized in Table 29.

**Figure 8:**
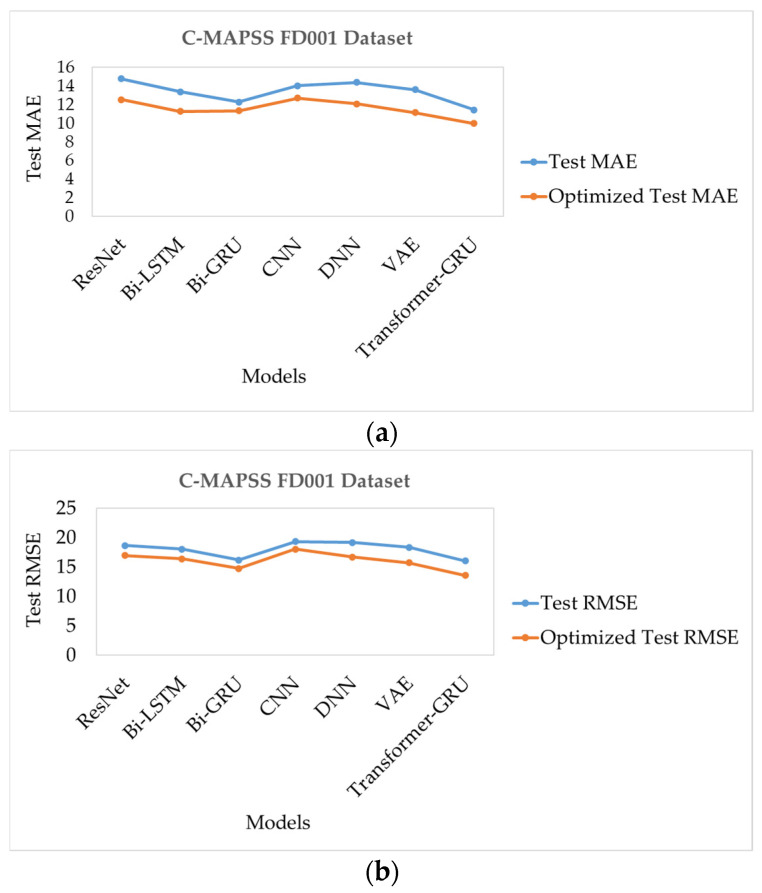
Error Performance Curves of different models on the C-MAPSS FD001 dataset before and after optimization: (**a**) MAE, (**b**) RMSE.

As shown in Table 29, Transformer-GRU achieves the best predictive performance, with an MAE of 11.45, an RMSE of 16.02, and a score of 410.1. Following optimization, Transformer-GRU further improves to an MAE of 9.97, an RMSE of 13.56, and a score of 254.3, demonstrating highly accurate predictions. Bi-GRU performs strongly, achieving an MAE of 12.28, an RMSE of 16.20, and a score of 484.9, which improves to 11.33, 14.78, and 330.8 after optimization. Bi-LSTM provides competitive results, with an MAE of 13.38, an RMSE of 18.05, and a score of 765.7, improving to 11.27, 16.41, and 661.0 after optimization. VAE and ResNet show moderate performance, with VAE achieving an MAE of 13.63, an RMSE of 18.34, and a score of 863.8, optimized to 11.16, 15.72, and 462.4, and ResNet recording an MAE of 14.79, an RMSE of 18.64, and a score of 826.4, optimized to 12.56, 16.97, and 761.5. CNN and DNN exhibit comparatively lower predictive performance, with CNN showing an MAE of 14.03, an RMSE of 19.32, and a score of 943.8, optimized to 12.70, 18.07, and 717.2, and DNN recording an MAE of 14.38, an RMSE of 19.20, and a score of 1779.5, optimized to 12.09, 16.71, and 610.1. Overall, Transformer-GRU is the most effective models for RUL prediction on the FD001 dataset, as shown in Figure 8a,b.

**Figure 9:**
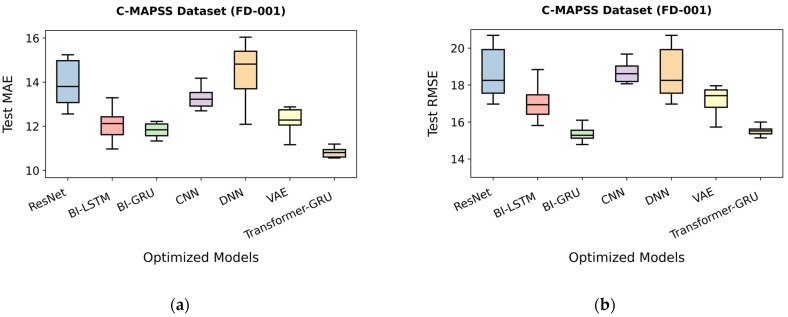
Distribution of optimized test errors for different models on the C-MAPSS FD001 dataset: (**a**) MAE, (**b**) RMSE.

The predictive performance of optimized models on the C-MAPSS FD001 dataset is presented in Figure 9a using boxplots of MAE values. Transformer-GRU achieves the lowest MAE among all models, indicating highly accurate predictions. Bi-GRU and Bi-LSTM follow closely, demonstrating competitive performance with moderate variability, while CNN, DNN, VAE, and ResNet show larger errors and wider distributions, reflecting comparatively lower prediction precision.

Figure 9b further illustrates the RMSE distributions of the models. Consistent with the MAE results, Transformer-GRU exhibits the lowest and most stable RMSE values, confirming robust predictive capability. Bi-GRU and Bi-LSTM again show satisfactory performance, whereas CNN, DNN, VAE, and ResNet display higher errors and variability. Overall, Transformer-GRU demonstrates superior performance and stability, highlighting its effectiveness in capturing complex temporal patterns in the dataset.

### 4.5. Performance of Predictive Models of C-MAPSS FD003 Dataset

In predictive maintenance tasks, accurate estimation of the remaining useful life (RUL) is critical for enhancing system reliability and reducing operational costs. Table 30 presents the prediction errors of various models on the C-MAPSS FD003 dataset, both before and after optimization.

**Figure 10:**
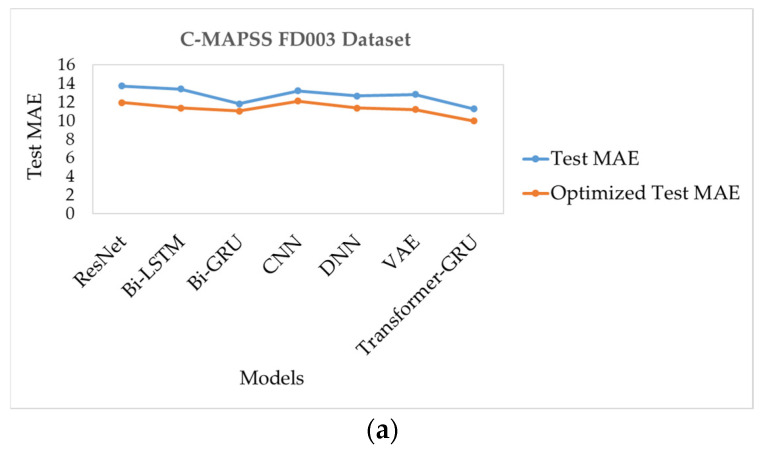

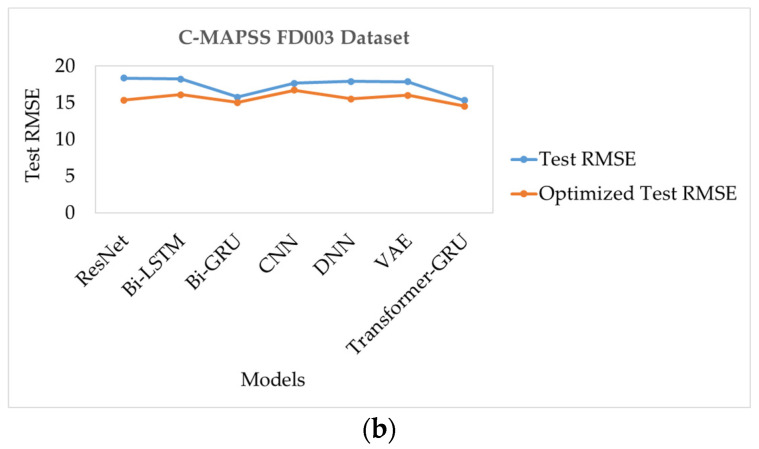
Error Performance Curves of different models on the C-MAPSS FD003 dataset before and after optimization: (**a**) MAE, (**b**) RMSE.

As shown in Table 30, the optimized Transformer-GRU model achieves the best overall performance, with a mean absolute error (MAE) of 9.98, a root mean square error (RMSE) of 14.57, and a Score of 320.8, indicating highly precise RUL predictions. The optimized Bi-GRU model also performs strongly, with an MAE of 11.04, an RMSE of 15.06, and a Score of 345.1. The optimized ResNet and DNN models show significant improvement after optimization, achieving MAE values of 11.97 and 11.39, RMSE values of 15.36 and 15.52, and Scores of 416.1 and 409.4, respectively. Similarly, optimized CNN reaches an MAE of 12.13, RMSE of 16.72, and a Score of 418.4, while the optimized Bi-LSTM model records an MAE of 11.39, RMSE of 16.11, and a Score of 469.9. The optimized VAE model demonstrates moderate improvements, with an MAE of 11.22, an RMSE of 16.03, and a Score of 495.9. Overall, optimization substantially reduces prediction errors across all models, with Transformer-GRU showing the most significant enhancement in predictive performance. These results are summarized in Figure 10a,b.

**Figure 11:**
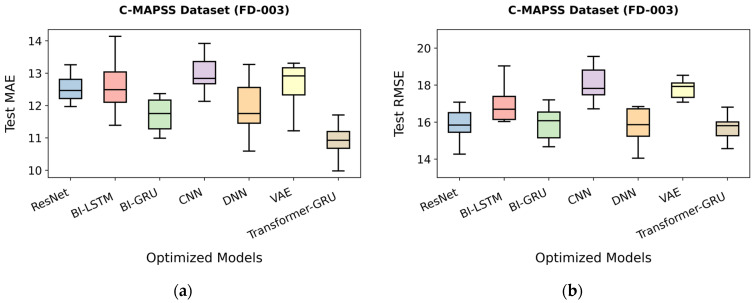
Distribution of optimized test errors for different models on the C-MAPSS FD003 dataset: (**a**) MAE, (**b**) RMSE.

The performance of the optimized models on the C-MAPSS FD-003 dataset is illustrated through Figure 11a,b of Test MAE and Test RMSE, providing insights into their predictive performance and consistency. Among the models, ResNet exhibits relatively low error values with moderate variability, reflecting its strong feature extraction capabilities for complex temporal patterns. BI-LSTM and BI-GRU show improved sequence modeling performance, with BI-GRU demonstrating slightly tighter error distributions, indicating more consistent predictions. CNN and DNN present larger error ranges, suggesting limitations in capturing long-term dependencies and degradation trends, while the VAE achieves moderate performance but higher variability due to its probabilistic reconstruction approach. The Transformer-GRU consistently achieves the lowest median errors and the narrowest distribution across both MAE and RMSE metrics, highlighting its superior ability to capture complex temporal dependencies and nonlinear degradation patterns. This consistent performance reflects both high predictive performance and robustness, underscoring the Transformer-GRU’s superior performance and stability across all test cases.

### 4.6. Fold Cross-Validation Results of the Optimized Transformer-GRU Model

A 5-fold cross-validation was conducted on the TTF, IMS, C-MAPSS FD001, and FD003 datasets using a restricted number of training epochs to rigorously evaluate the stability and reproducibility of the Transformer-GRU model while ensuring that training and testing splits were devoid of data leakage. This limited training regime was designed to assess high-level model consistency instead of achieving minimal predictive error. In accordance with experimental rationale, fold-wise MAE and RMSE values are higher than those from fully trained models; however, predictions remain highly consistent across folds, demonstrating the model’s robust generalization capability. These results confirm that the Transformer-GRU reliably captures intrinsic temporal and feature dependencies even under a restricted training regime. Moreover, the observed trends in Table 31 align with the fully optimized model results (Table 27, Table 28, Table 29 and Table 30), validating that the reported cross-validation performance reflects stable and reproducible model behavior rather than degradation of predictive ability.

### 4.7. Computational Cost of Hybrid HHO–WHO Algorithm and Time, and Memory for Optimized Transformer-GRU Model

The computational efficiency and resource utilization of the Transformer-GRU model were evaluated after applying the hybrid Harris Hawks Optimization–Wild Horse Optimization Algorithm (HHO–WHO) across multiple benchmark datasets. The analysis includes the computational cost of the optimization process, encompassing population size, number of iterations, total fitness evaluations, hyperparameter encoding, and stopping criteria. Training and inference times of optimized model were measured per batch and per sample to assess the model’s speed and real-time prediction capability. Memory consumption of optimized model was analyzed to determine the model’s suitability for resource-constrained environments. These results demonstrate that the Transformer-GRU model achieves rapid training, efficient inference, and low memory usage, confirming its applicability for industrial predictive maintenance tasks.

#### 4.7.1. Computational Cost of Hybrid HHO–WHO Algorithm

The Transformer-GRU fault prediction model was optimized using a hybrid Harris Hawks Optimization–Wild Horse Optimization Algorithm HHO–WHO. As detailed in Table 32, The optimizer used a population size of 10 candidate solutions, allowing sufficient exploration of the hyperparameter space while maintaining computational efficiency. The algorithm ran for 20 generations, resulting in 200 total fitness evaluations, as each candidate was evaluated once per generation. Each evaluation involved training the model for 50 epochs with a batch size of 64, which affects both training stability and the computational cost per evaluation. Hyperparameters were encoded using a mixed scheme: continuous variables such as learning rate and dropout rate, and discrete variables including GRUs, dense units, attention heads, GRU layers, and dense layers, which were rounded or clipped during optimization. The stopping criterion was defined as a fixed number of generations 20, ensuring full reproducibility of the optimization process. 

#### 4.7.2. Optimized Model Training Time

The training time of the Transformer-GRU model is evaluated on four benchmark datasets to analyze its computational efficiency after applying the HHO–WHO algorithm, as shown in Table 33. For the TTF dataset, the model requires 0.0960 s per batch and 0.75 milliseconds (ms) per sample, indicating very fast training performance. On the IMS dataset, the training time per batch is 0.1063 s with a per sample time of 0.83 ms, reflecting a slight increase due to dataset complexity. The C-MAPSS FD001 dataset exhibits the longest training time of 0.1266 s per batch and 0.98 ms per sample, attributed to its larger size and more complex features. For the C-MAPSS FD003 dataset, the model records 0.1200 s per batch and 0.93 ms per sample, maintaining efficient processing despite the dataset’s complexity. These results demonstrate that the Transformer-GRU model achieves consistent and optimized training times across all datasets, confirming its capability to provide rapid and reliable model learning suitable for predictive maintenance tasks while maintaining computational efficiency.

#### 4.7.3. Optimized Model Inference Time

The inference time of the optimized Transformer-GRU model is evaluated on four benchmark datasets to assess its real-time prediction efficiency. As shown in Table 34, the model achieves an inference time per batch of 0.0799 s and per sample of 0.62 ms on the TTF dataset. On the IMS dataset, the inference time per batch increases to 0.0886 s and per sample to 0.69 ms. For the C-MAPSS FD001 dataset, the model records the longest inference time per batch of 0.1030 s and per sample of 0.80 ms. The C-MAPSS FD003 dataset exhibits an inference time per batch of 0.1023 s and per sample of 0.79 ms. The Transformer-GRU model demonstrates efficient and consistent inference performance across all datasets, indicating its suitability for real-time predictive maintenance applications.

#### 4.7.4. Optimized Model Memory Consumption

The memory consumption of the Transformer-GRU model is analyzed across four benchmark datasets to evaluate its computational efficiency after applying the HHO–WHO optimization algorithm, as shown in Table 35. On the TTF dataset, the model consumes 0.97 MB of memory, indicating minimal resource utilization. For the IMS dataset, memory usage increases to 1.34 MB, reflecting more complex input features. The C-MAPSS FD001 dataset requires 1.38 MB of memory, while the C-MAPSS FD003 dataset consumes 1.39 MB, demonstrating that memory requirements scale moderately with dataset complexity. These results confirm that the Transformer-GRU model maintains low memory consumption across all datasets, making it highly suitable for predictive maintenance tasks where computational resources are limited, and its optimized training and inference times are efficiently supported across all datasets.

#### 4.7.5. Energy Efficiency

Energy efficiency is a critical consideration when deploying deep learning models for failure prediction in industrial machinery and engine, especially on resource-constrained platforms such as edge devices and embedded systems. Total energy consumption E quantifies the overall power required to perform computational tasks, while energy efficiency η reflects the effectiveness of converting this energy into useful computational output. Energy consumption is largely influenced by algorithmic complexity, hardware architecture, and model implementation [73]. It can be estimated as:

(16) $$E=\mu *CC*{\left(F\right)}^{2}$$

where μ is a proportionality constant related to chip capacitance, CC is the total clock cycles, and F is the processor frequency. Correspondingly, energy efficiency is defined as [74]:

(17) $$\eta =\frac{Performance}{E}$$

With *Performance* measured via model evaluation metrics such as performance or detection rate. Enhancing energy efficiency involves reducing energy consumption (*E*) while maintaining or improving performance, using strategies such as pruning redundant parameters, applying quantization, and utilizing low-power hardware accelerators. These practices enable efficient deployment of high-performing predictive models in energy-sensitive industrial environments.

### 4.8. Comparison Experiments

The predictive performance of the proposed Transformer-GRU model optimized using the HHO–WHO algorithm is benchmarked against several state-of-the-art methods across the TTF, IMS, and C-MAPSS datasets. As shown in Table 36, the proposed approach consistently delivers the lowest prediction errors.

For the TTF dataset, prior models such as TCN, AE, DNN, MLP, CNN, GRU, and LSTM report MAE values of 2.560, 1.840, 1.620, 1.440, 0.930, 0.870, and 0.830, respectively, with corresponding RMSE values ranging from 1.52 to 3.97 [32]. In contrast, the proposed Transformer-GRU with HHO–WHO achieves a significantly lower MAE of 0.152 and RMSE of 0.23, demonstrating a substantial improvement in predictive performance.

For the IMS dataset, prior SVM-based models including GSA-SVM, PSO-SVM, GA-SVM, and SOA-SVM report MAE values ranging from 0.0576 to 0.0625 and RMSE values between 0.0783 and 0.0911 [43]. Additionally, 1D-CNN models across Bearings 1–4 exhibit MAE values between 0.0462 and 0.0980, with RMSE values from 0.0691 to 0.1180 [75]. The proposed method attains an overall MAE of 0.0126 and RMSE of 0.0242, confirming its robustness under varying operating conditions and noise levels.

On the C-MAPSS FD001 dataset, existing approaches such as PSO-1DCNN, GA-1DCNN, GWO-1DCNN, BO-LSTM, Improved GWO-1DCNN, CNN, DeepLSTM, DBN, and CNN-LSTM exhibit MAE values from 10.14 to 16.12 and RMSE values from 13.76 to 18.45, with Scores ranging from 338.0 to 1290 [76,77]. The proposed model reduces the MAE to 9.97, RMSE to 13.56, and Score to 254.3, highlighting its capability to capture nonlinear degradation patterns in engine systems.

Similarly, for C-MAPSS FD003, prior methods record MAE values between 12.10 and 17.27, RMSE values between 14.71 and 19.82, and Scores from 442.0 to 1596 [76,77]. Notably, the Improved GWO-1DCNN achieves MAE of 12.10, RMSE of 15.51, and Score of 608.3. The proposed Transformer-GRU with HHO–WHO achieves the lowest MAE of 9.98, RMSE of 14.57, and Score of 320.8, again demonstrating consistent superiority across more complex operating conditions.

The results validate that the proposed Transformer-GRU architecture, enhanced through hybrid HHO–WHO optimization, consistently outperforms existing approaches. Its high predictive performance, low MAE and RMSE, and competitive Score across multiple datasets underscore its suitability as a scalable and reliable solution for remaining useful life prediction and failure time estimation in industrial prognostics applications.

### 4.9. Ablation Experiments

The ablation study presented in Table 37 provides a comprehensive evaluation of the individual and combined contributions of WHO, HHO, and the hybrid HHO–WHO optimization strategies to the predictive performance of the Transformer-GRU architecture across the TTF, IMS, and C-MAPSS datasets. The baseline Transformer-GRU configuration demonstrates moderate predictive capability, yielding MAE and RMSE values of 0.718 and 1.313 on TTF, 0.036 and 0.059 on IMS, 11.45 and 16.02 on C-MAPSS FD001, and 11.28 and 15.33 on C-MAPSS FD003.

The introduction of WHO results in consistent improvements, reducing MAE and RMSE to 0.622 and 1.080 for TTF, 0.032 and 0.060 for IMS, 10.68 and 14.34 for FD001, and 11.17 and 15.27 for FD003. These improvements reflect the enhanced local search capability provided by WHO, which mitigates premature convergence. Subsequent integration of HHO further strengthens model performance, providing a more balanced exploration and exploitation trade-off and contributing to additional reductions in prediction errors, particularly for TTF with MAE 0.370 and RMSE 0.960 and IMS with MAE 0.022 and RMSE 0.039, while maintaining competitive results for FD001 with MAE 10.98 and RMSE 15.22 and FD003 with MAE 10.70 and RMSE 14.90.

The hybrid HHO–WHO optimization achieves the most substantial performance gains. For TTF, MAE decreases to 0.152 and RMSE to 0.232, while IMS achieves MAE 0.013 and RMSE 0.024, demonstrating robustness under variable load conditions and sensor noise. In C-MAPSS, the hybrid model reduces MAE to 9.97 for FD001 and 9.98 for FD003, with corresponding RMSE values of 13.56 and 14.57, and improves the overall C-MAPSS Score to 254.3 for FD001 and 320.8 for FD003. These results consistently confirm the superiority of the hybrid optimization over standalone WHO and HHO strategies.

The hybridization scheme systematically partitions candidate solutions into two complementary subsets based on even and odd indices. Candidates at even indices are updated using HHO, emphasizing global exploration, while candidates at odd indices are updated using WHO, emphasizing local exploitation. This structured allocation enables parallel and complementary search behaviors, enhancing diversity in the solution space while maintaining directed convergence. The hybridization scheme systematically balances exploration and exploitation across complementary candidate subsets, yielding consistent improvements and demonstrating a principled approach.

The findings demonstrate that the hybrid HHO–WHO framework delivers complementary optimization advantages that significantly enhance the Transformer-GRU model’s ability to capture temporal dependencies and nonlinear degradation behaviors. The consistent improvements across TTF, IMS, and C-MAPSS validate the effectiveness, robustness, and generalizability of the proposed approach, positioning it as a strong candidate for reliable remaining useful life prediction and fault prognostics within industrial monitoring environments.

## 5. Challenges and Limitations

A primary challenge in failure time (FT) and remaining useful life (RUL) prediction is the extensive manual effort required for hyperparameter tuning in deep learning models. This process is often time-consuming and computationally expensive and limits scalability in real-world industrial applications. Furthermore, model generalization across datasets with varying operational conditions remains a critical challenge. Many models perform well in controlled environments but exhibit poor adaptability when exposed to new, unseen data, undermining their practical deployment in diverse industrial scenarios. Another significant challenge is effectively capturing temporal dependencies and complex degradation patterns inherent in industrial machinery and engine data. The dynamic, nonlinear, and multi-scale nature of failure patterns demands advanced modeling capabilities, which many conventional approaches fail to provide. These challenges collectively highlight the need for an intelligent, automated, and robust optimization strategy. The proposed hybrid HHO–WHO framework leverages HHO’s global exploration to search broadly across the hyperparameter space and WHO’s local exploitation to efficiently converge toward optimal solutions, thus balancing exploration and exploitation, mitigating local optima entrapment, and improving generalization. When coupled with an optimized Transformer-GRU architecture, this framework enhances predictive performance, robustness, and scalability across diverse industrial datasets, including TTF, IMS, and C-MAPSS FD001 and FD003. It provides a reliable and adaptive solution for real-world predictive maintenance, addressing limitations of manual tuning, insufficient temporal modeling, and poor model generalization. Despite addressing key issues in predictive failures, the proposed framework still exhibits certain limitations that require further investigation:

- Optimizing additional deep learning parameters using more advanced hybrid optimization techniques may further improve model performance and adaptability.
- Validating the framework across a broader range of machine and engine types and industrial conditions is essential to increase its generalizability and practical deployment.

## 6. Conclusions

This study presents a novel hybrid optimization framework that combines HHO and WHO algorithms to automatically fine-tune the hyperparameters of multiple predictive models, including ResNet, Bi-LSTM, Bi-GRU, CNN, DNN, VAE, and Transformer-GRU. The framework leverages HHO for global exploration and WHO for local exploitation, achieving a balanced search that mitigates local optima entrapment and improves generalization. The models were evaluated on four benchmark datasets, including TTF, IMS, C-MAPSS FD001, and FD003, representing diverse industrial degradation scenarios. Among all models, the hybrid HHO–WHO optimized Transformer-GRU consistently achieved the most significant improvement in predictive performance. On the TTF dataset, MAE decreased from 0.72 to 0.15 and RMSE from 1.31 to 0.23. On the IMS dataset, MAE dropped from 0.04 to 0.01 and RMSE from 0.06 to 0.02. On C-MAPSS FD001, MAE decreased from 11.45 to 9.97 9.63, RMSE from 16.02 to 13.56, and score from 410.1 to 254.3. On C-MAPSS FD003, MAE decreased from 11.28 to 9.98, RMSE from 15.33 to 14.57, and score from 352.3 to 320.8. Furthermore, the approach was validated through comparison and ablation studies, confirming the robustness, generalization, and overall effectiveness of the proposed hybrid framework. These results demonstrate that the Transformer-GRU model optimized via HHO–WHO provides a reliable, stable and adaptive solution for predictive maintenance, consistently outperforming conventional baseline models. The proposed hybrid framework establishes a benchmark for high-performance failure time and RUL forecasting in complex industrial environments, offering an effective and practical tool for minimizing unplanned downtime and enhancing maintenance decision-making.

Future work will focus on optimizing additional deep learning parameters using advanced hybrid optimization techniques to further enhance model adaptability and performance. The proposed framework will be validated across a wider range of machine and engine types and real-world industrial conditions to improve generalizability, scalability, and support practical deployment. Expanding application domains and operational scenarios will confirm the framework’s robustness and facilitate its implementation in real-world predictive maintenance systems, while being extended to streaming and online settings to enable real-time adaptability. Moreover, direct empirical evaluation of energy consumption under standardized hardware and execution protocols will be conducted to assess the energy efficiency of the proposed model.

## Figures and Tables

**Table 1:** ResNet model structure before optimization.

Section	Layer Type	Filters/Units	Activation Function
Input Stage	Conv1D	32	ReLU
Normalization	BatchNormalization	-	-
Residual Block 1	Conv1D (×2) + Add	32	ReLU
Residual Block 2	Conv1D (×2) + Add	32	ReLU
Pooling	GlobalAveragePooling1D	-	-
Regularization	Dropout	-	-
Hidden Stage	Dense	16	ReLU
Output Stage	Dense	1	Linear

**Table 2:** Hyperparameter configuration of the ResNet model before optimization.

Setting	Specification
Batch Processing Size	64
Learning Rate	0.02
Optimization Algorithm	Adam
Loss Function	MAE
Regularization Dropout	0.01
Performance Metrics	MAE, RMSE

**Table 3:** Bi-LSTM model structure before optimization.

Section	Layer Type	Units/Features	Activation Function
Input Stage	Bidirectional LSTM	32	Tanh
Regularization	Dropout	-	-
Hidden Stage	Fully Connected	16	ReLU
Output Stage	Fully Connected	1	Linear

**Table 4:** Hyperparameter configuration of the Bi-LSTM model before optimization.

Setting	Specification
Batch Processing Size	64
Learning Rate	0.02
Optimization Algorithm	Adam
Loss Function	MAE
Regularization Dropout	0.1
Performance Metrics	MAE, RMSE

**Table 5:** Bi-GRU model structure before optimization.

Section	Layer Type	Units/Features	Activation Function
Input Stage	Bidirectional GRU	64	Tanh
Regularization	Dropout	-	-
Hidden Stage	Fully Connected	32	ReLU
Output Stage	Fully Connected	1	Linear

**Table 6:** Hyperparameter configuration of the Bi-GRU model before optimization.

Setting	Specification
Batch Processing Size	64
Learning Rate	0.001
Optimization Algorithm	Adam
Loss Function	MAE
Regularization Dropout	0.1
Performance Metrics	MAE, RMSE

**Table 7:** CNN model structure before optimization.

Section	Layer Type	Units/Features	Activation Function
Input Stage	Conv1D	16 filters, kernel size 5	ReLU
Dimensionality Reduction	MaxPooling1D	Pool size 2	-
Feature Transformation	Dense	64	ReLU
Output Stage	Dense	1	Linear

**Table 8:** Hyperparameter configuration of the CNN model before optimization.

Setting	Specification
Batch Size	64
Learning Rate	0.001
Optimization Algorithm	Adam
Loss Function	MAE
Performance Metrics	MAE, RMSE

**Table 9:** DNN model structure before optimization.

Section	Layer Type	Units/Features	Activation Function
Input Layer	Dense	64	ReLU
Regularization Layer	Dropout	0.05	-
Hidden Layer	Dense	64	ReLU
Regularization Layer	Dropout	0.05	-
Output Layer	Dense	1	Linear

**Table 10:** Hyperparameter configuration of the DNN model before optimization.

Setting	Specification
Batch Size	64
Learning Rate	0.01
Optimization Algorithm	Adam
Loss Function	MAE
Dropout Rate	0.05
Evaluation Metrics	MAE, RMSE

**Table 11:** VAE model structure before optimization.

Section	Layer Type	Units/Features	Activation Function
Encoder Layer 1	Dense	256	ReLU
Encoder Layer 2	Dense	128	ReLU
Encoder Regularization	Dropout	0.4	-
Latent Representation	Dense	32	Linear (z_mean/z_log_var)
Decoder Layer 1	Dense	128	ReLU
Decoder Layer 2	Dense	256	ReLU
Decoder Regularization	Dropout	0.4	-
Decoder Output Layer	Dense	4	Linear
Regression Layer 1	Dense	256	ReLU
Regression Regularization	Dropout	0.4	-
Regression Layer 2	Dense	256	ReLU
Regression Layer 3	Dense	128	ReLU
Regression Layer 4	Dense	64	ReLU
Regression Output Layer	Dense	1	Linear

**Table 12:** Hyperparameter configuration of the VAE model before optimization.

Setting	Specification
Batch Size	32
Learning Rate	0.0001
Optimization Algorithm	Adam
Loss Function	MSE (for regression), VAE loss = reconstruction + KL
Dropout Rate	0.4
Latent Dimension	32
Evaluation Metrics	MAE, RMSE

**Table 13:** Transformer-GRU model structure before optimization.

Section	Layer Type	Units/Features	Activation Function
Input Layer	Input	N features × 1	-
Transformer Block 1	Multi-Head Attention	2 heads, key dim 64	-
	Layer Normalization	-	-
	Add/Residual	-	-
	Dense (FFN)	128	ReLU
	Dense (FFN)	128	Linear
	Add/Residual	-	-
	Layer Normalization	-	-
Transformer Block 2	Multi-Head Attention	2 heads, key dim 64	-
	Layer Normalization	-	-
	Add/Residual	-	-
	Dense (FFN)	128	ReLU
	Dense (FFN)	128	Linear
	Add/Residual	-	-
	Layer Normalization	-	-
Temporal Layer	GRU	256	-
Regularization Layer	Dropout	0.05	-
Feature Transformation	Dense	64	ReLU
Output Layer	Dense	1	Linear

**Table 14:** Hyperparameter configuration of the Transformer-GRU model before optimization.

Setting	Specification
Batch Size	64
Learning Rate	0.001
Optimization Algorithm	Adam
Loss Function	MAE
Dropout Rate	0.05
GRUs	256
Attention Heads	2
Evaluation Metrics	MAE, RMSE

**Table 15:** Optimization ranges for all evaluated models using hybrid HHO–WHO algorithm.

Model	Parameter	Type	Range
ResNet	Learning Rate	Continuous	range (1 × 10^−5^, 1 × 10^−2^)
	ResNet Filters	Discrete	range (16, 128)
	Dense Units	Discrete	range (16, 128)
	Dropout Rate	Continuous	range (0.0, 0.5)
Bi-LSTM	Learning Rate	Continuous	range (1 × 10^−5^, 1 × 10^−2^)
	LSTM Units	Discrete	range (16, 128)
	Dense Units	Discrete	range (16, 128)
	Dropout Rate	Continuous	range (0.0, 0.5)
Bi-GRU	Learning Rate	Continuous	range (1 × 10^−5^, 1 × 10^−2^)
	GRUs	Discrete	range (16, 128)
	Dense Units	Discrete	range (16, 128)
	Dropout Rate	Continuous	range (0.0, 0.5)
CNN	Learning Rate	Continuous	range (1 × 10^−5^, 0.1)
	Filters	Discrete	range (8, 128)
	Kernel Size	Discrete	range (2, 5)
	Dense Units	Discrete	range (16, 256)
DNN	Learning Rate	Continuous	range (1 × 10^−5^, 0.1)
	Dense Units	Discrete	range (16, 256)
	Dropout Rate	Continuous	range (0.0, 0.5)
	Number of Layers	Discrete	range (1, 4)
VAE	Learning Rate	Continuous	range (1 × 10^−4^, 5 × 10^−3^)
	Hidden Units	Discrete	range (16, 2048)
	Dropout Rate	Continuous	range (0.0, 0.3)
Transformer-GRU	Learning Rate	Continuous	range (1 × 10^−5^, 0.1)
	GRUs	Discrete	range (16, 512)
	Dense Units	Discrete	range (16, 512)
	Dropout Rate	Continuous	range (0.0, 0.8)
	Attention Heads	Discrete	range (1, 12)
	GRU Layers	Discrete	range (1, 7)
	Dense Layers	Discrete	range (1, 7)

**Table 16:** Statistical analysis of TTF dataset.

Dataset	TTF
Samples	18,566
Features	4
Output Variable	Failure time
Min	0
Max	580
Mean	54.91
Std	96.63

**Table 17:** Statistical analysis of IMS dataset.

Dataset	IMS
Samples	3936
Features	48
Output Variable	RUL
Min	1150
Max	2192
Mean	1667.75
Std	284.88

**Table 18:** Statistical characteristics of the FD001 subset of the C-MAPSS dataset.

Dataset	C-MAPSS (FD001)
Samples	20,631
Features	25
Output Variable	RUL
Min	0
Max	125
Mean	86.83
Std	41.67

**Table 19:** Statistical characteristics of the FD003 subset of the C-MAPSS dataset.

Dataset	C-MAPSS (FD003)
Samples	24,720
Features	25
Output Variable	RUL
Min	0
Max	125
Mean	93.14
Std	40.63

**Table 20:** HHO–WHO values for ResNet parameters.

Parameter	Value
Model	ResNet
Learning Rate	0.0015
ResNet Filters	41
Dense Units	19
Dropout Rate	0.03

**Table 21:** HHO–WHO values for BI-LSTM parameters.

Parameter	Value
Model	BI-LSTM
Learning Rate	0.0053
LSTM Units	59
Dense Units	26
Dropout Rate	0.01

**Table 22:** HHO–WHO values for BI-GRU parameters.

Parameter	Value
Model	BI-GRU
Learning Rate	0.0024
GRUs	44
Dense Units	16
Dropout Rate	0.0

**Table 23:** HHO–WHO values for CNN parameters.

Parameter	Value
Model	CNN
Learning Rate	0.0019
Filters	37
Kernel Size	3
Dense Units	188

**Table 24:** HHO–WHO values for DNN parameters.

Parameter	Value
Model	DNN
Learning Rate	0.0044
Dense Units	80
Dropout Rate	0.0
Number of Layers	1

**Table 25:** HHO–WHO values for VAE parameters.

Parameter	Value
Model	VAE
Hidden Units	1024
Dropout Rate	0.1
Learning Rate	0.001

**Table 26:** HHO–WHO values for Transformer-GRU parameters.

Parameter	Value
Model	Transformer-GRU
Learning Rate	0.00094
GRUs	44
Dense Units	16
Dropout Rate	0.0
Attention Heads	3
GRU Layers	2
Dense Layers	1

**Table 27:** Prediction errors of different models on the TTF dataset.

Model	MAE	RMSE	Optimized MAE	Optimized RMSE
ResNet	4.4839	5.4856	1.5829	2.7382
Bi-LSTM	2.9447	4.5280	1.3248	1.9440
Bi-GRU	2.0314	2.7875	1.0262	1.4911
CNN	1.6162	3.3811	0.6721	1.2916
DNN	2.1766	3.9286	1.8388	3.6629
VAE	3.6098	4.9181	0.8208	1.3714
Transformer-GRU	0.7176	1.3131	0.1516	0.2317

**Table 28:** Prediction errors of different models on the IMS dataset.

Model	MAE	RMSE	Optimized MAE	Optimized RMSE
ResNet	0.0836	0.1134	0.0267	0.0328
Bi-LSTM	0.1149	0.1556	0.0819	0.1134
Bi-GRU	0.0867	0.1143	0.0565	0.0871
CNN	0.1011	0.1305	0.0834	0.1198
DNN	0.1037	0.1340	0.0926	0.1273
VAE	0.1611	0.2211	0.1487	0.1974
Transformer-GRU	0.0356	0.0586	0.0126	0.0242

**Table 29:** Prediction errors of different models on the C-MAPSS FD001 dataset.

Model	MAE	RMSE	Score	Optimized MAE	Optimized RMSE	Optimized Score
ResNet	14.79	18.64	826.4	12.56	16.97	761.5
Bi-LSTM	13.38	18.05	765.7	11.27	16.41	661.0
Bi-GRU	12.28	16.20	484.9	11.33	14.78	330.8
CNN	14.03	19.32	943.8	12.70	18.07	717.2
DNN	14.38	19.20	1779.5	12.09	16.71	610.1
VAE	13.63	18.34	863.8	11.16	15.72	462.4
Transformer-GRU	11.45	16.02	410.1	9.97	13.56	254.3

**Table 30:** Prediction errors of different models on the C-MAPSS FD003 dataset.

Model	MAE	RMSE	Score	Optimized MAE	Optimized RMSE	Optimized Score
ResNet	13.73	18.37	703.3	11.97	15.36	416.1
Bi-LSTM	13.42	18.26	644.1	11.39	16.11	469.9
Bi-GRU	11.83	15.79	394.9	11.04	15.06	345.1
CNN	13.21	17.69	642.7	12.13	16.72	418.4
DNN	12.67	17.94	610.4	11.39	15.52	409.4
VAE	12.84	17.88	504.4	11.22	16.03	495.9
Transformer-GRU	11.28	15.33	352.3	9.98	14.57	320.8

**Table 31:** 5-Fold Cross-Validation Results for the Optimized Transformer-GRU Model.

Dataset	Average MAE	Average RMSE
TTF	0.4978	0.7309
IMS	0.0338	0.0566
C-MAPSS (FD001)	13.7822	18.8141
C-MAPSS (FD003)	14.0638	19.0970

**Table 32:** Detailed computational cost metrics of HHO–WHO algorithm.

Parameter	Value
Population Size	10
Number of Generations/Iterations	20
Total Fitness Evaluations	200
Stopping Criteria	Fixed number of generations 20
Hyperparameter Encoding	Mixed continuous and discrete
Training Epochs per Evaluation	50
Batch Size	64

**Table 33:** Training time for Transformer-GRU Model after applying HHO–WHO algorithm.

Dataset	Training Time per Batch (s)	Training Time per Sample (ms)
TTF	0.0960	0.75
IMS	0.1063	0.83
C-MAPSS (FD001)	0.1266	0.98
C-MAPSS (FD003)	0.1200	0.93

**Table 34:** Inference time for Transformer-GRU Model after applying HHO–WHO algorithm.

Dataset	Inference Time per Batch (s)	Inference Time per Sample (ms)
TTF	0.0799	0.62
IMS	0.0886	0.69
C-MAPSS (FD001)	0.1030	0.80
C-MAPSS (FD003)	0.1023	0.79

**Table 35:** Memory consumption for Transformer-GRU Model after applying HHO–WHO algorithm.

Dataset	Memory Consumption (MB)
TTF	0.97
IMS	1.34
C-MAPSS (FD001)	1.38
C-MAPSS (FD003)	1.39

**Table 36:** Results of comparison experiments of prior methods and proposed method across TTF, IMS, and C-MAPSS datasets.

Dataset	Model	MAE	RMSE	Score
TTF	TCN [32]	2.560	3.97	-
	AE [32]	1.840	2.71	-
	DNN [32]	1.620	2.6	-
	MLP [32]	1.440	1.89	-
	CNN [32]	0.930	1.52	-
	GRU [32]	0.870	1.79	-
	LSTM [32]	0.830	1.62	-
	HHO–WHO + Transformer-GRU (Proposed)	0.152	0.23	-
IMS	GSA-SVM [43]	0.0576	0.0783	-
	PSO-SVM [43]	0.0606	0.0890	-
	GA-SVM [43]	0.0620	0.0907	-
	SOA-SVM [43]	0.0625	0.0911	-
	1D-CNN (Bearing 1) [75]	0.0462	0.0691	-
	1D-CNN (Bearing 2) [75]	0.0980	0.1180	-
	1D-CNN (Bearing 3) [75]	0.0868	0.1071	-
	1D-CNN (Bearing 4) [75]	0.0677	0.0822	-
	HHO–WHO + Transformer-GRU (Proposed, overall)	0.0126	0.0242	-
C-MAPSS FD001	PSO-1DCNN [76]	10.84	14.65	493.6
	GA-1DCNN [76]	11.37	15.13	554.3
	GWO-1DCNN [76]	11.06	14.71	531.6
	BO-LSTM [76]	14.02	15.19	611.3
	Improved GWO-1DCNN [76]	10.14	13.76	462.9
	CNN [77]	16.12	18.45	1290
	DeepLSTM [77]	14.58	16.14	338.0
	DBN [77]	13.46	15.21	418.0
	CNN-LSTM [76]	12.24	14.82	526.4
	HHO–WHO + Transformer-GRU (Proposed)	9.97	13.56	254.3
C-MAPSS FD003	PSO-1DCNN [76]	12.68	15.97	672.4
	GA-1DCNN [76]	13.10	16.80	780.6
	GWO-1DCNN [76]	12.91	16.36	730.9
	BO-LSTM [76]	14.52	17.02	851.2
	Improved GWO-1DCNN [76]	12.10	15.51	608.3
	CNN [77]	17.27	19.82	1596
	DeepLSTM [77]	14.37	16.18	852.0
	DBN [77]	12.74	14.71	442.0
	CNN-LSTM [76]	14.09	16.83	681.4
	HHO–WHO + Transformer-GRU (Proposed)	9.98	14.57	320.8

**Table 37:** Results of ablation experiments.

Experimental Method	TTF		IMS		C-MAPSS (FD001)			C-MAPSS (FD003)		
	MAE	RMSE	MAE	RMSE	MAE	RMSE	Score	MAE	RMSE	Score
Transformer-GRU	0.718	1.313	0.036	0.059	11.45	16.02	410.1	11.28	15.33	352.3
WHO + Trans.-GRU	0.622	1.080	0.032	0.060	10.68	14.34	324.9	11.17	15.27	340.8
HHO + Trans.-GRU	0.370	0.960	0.022	0.039	10.98	15.22	387.0	10.70	14.90	329.8
HHO–WHO + Trans.-GRU (Proposed method)	0.152	0.232	0.013	0.024	9.97	13.56	254.3	9.98	14.57	320.8

## Data Availability

The raw data supporting the conclusions of this article for the TTF dataset will be made available by the authors upon reasonable request. The IMS Bearing Dataset can be downloaded publicly from its official website. The C-MAPSS Jet Engine Simulated dataset can be downloaded publicly from its official website.
